# Multi-omics of the esophageal microenvironment identifies signatures associated with progression of Barrett’s esophagus

**DOI:** 10.1186/s13073-021-00951-6

**Published:** 2021-08-19

**Authors:** Nandan P. Deshpande, Stephen M. Riordan, Claire J. Gorman, Shaun Nielsen, Tonia L. Russell, Carolina Correa-Ospina, Bentotage S. M. Fernando, Shafagh A. Waters, Natalia Castaño-Rodríguez, Si Ming Man, Nicodemus Tedla, Marc R. Wilkins, Nadeem O. Kaakoush

**Affiliations:** 1 School of Biotechnology and Biomolecular Sciences, UNSW Sydney, Sydney, NSW 2052 Australia; 2 Gastrointestinal and Liver Unit, The Prince of Wales Hospital, Randwick, NSW 2031 Australia; 3 School of Medical Sciences, Faculty of Medicine, UNSW Sydney, Sydney, NSW 2052 Australia; 4 Ramaciotti Centre for Genomics, UNSW Sydney, Sydney, NSW 2052 Australia; 5 School of Women’s and Children’s Health, UNSW Sydney, Sydney, NSW 2052 Australia; 6 The John Curtin School of Medical Research, The Australian National University, Canberra, ACT 2601 Australia

**Keywords:** Esophagus, Metaplasia, Adenocarcinoma, Microbiome, Transcriptome, *Campylobacter*, intracellular survival

## Abstract

**Background:**

The enrichment of Gram-negative bacteria of oral origin in the esophageal microbiome has been associated with the development of metaplasia. However, to date, no study has comprehensively assessed the relationships between the esophageal microbiome and the host.

**Methods:**

Here, we examine the esophageal microenvironment in gastro-esophageal reflux disease and metaplasia using multi-omics strategies targeting the microbiome and host transcriptome, followed by targeted culture, comparative genomics, and host-microbial interaction studies of bacterial signatures of interest.

**Results:**

Profiling of the host transcriptome from esophageal mucosal biopsies revealed profound changes during metaplasia. Importantly, five biomarkers showed consistent longitudinal changes with disease progression from reflux disease to metaplasia. We showed for the first time that the esophageal microbiome is distinct from the salivary microbiome and the enrichment of *Campylobacter* species as a consistent signature in disease across two independent cohorts. Shape fitting and matrix correlation identified associations between the microbiome and host transcriptome profiles, with a novel co-exclusion relationship found between *Campylobacter* and napsin B aspartic peptidase. Targeted culture of *Campylobacter* species from the same cohort revealed a subset of isolates to have a higher capacity to survive within primary human macrophages. Comparative genomic analyses showed these isolates could be differentiated by specific genomic features, one of which was validated to be associated with intracellular fitness. Screening for these *Campylobacter* strain-specific signatures in shotgun metagenomics data from another cohort showed an increase in prevalence with disease progression. Comparative transcriptomic analyses of primary esophageal epithelial cells exposed to the *Campylobacter* isolates revealed expression changes within those infected with strains with high intracellular fitness that could explain the increased likelihood of disease progression.

**Conclusions:**

We provide a comprehensive assessment of the esophageal microenvironment, identifying bacterial strain-specific signatures with high relevance to progression of metaplasia.

**Supplementary Information:**

The online version contains supplementary material available at 10.1186/s13073-021-00951-6.

## Background

Esophageal cancer is the 7th most common cancer and the 6th most common cause of cancer-related mortality [1]. There are two major subtypes of esophageal cancer, squamous cell carcinoma (ESCC) and adenocarcinoma (EAC), with levels of the former decreasing while those of the latter increasing globally [1, 2]. Survival of patients appears to be improving with time; however, 5-year survival rates remain very poor due to late diagnosis [1, 2].

There are well-established risk factors for the development of EAC, including reflux symptoms, obesity, sex, age, and ethnicity [3]. The chronic exposure to refluxate of acid and bile leading to gastro-esophageal reflux disease (GERD) is the strongest known risk factor [3]. Long-term GERD leads to metaplastic changes, termed Barrett’s esophagus (BAR), a known precursor of EAC [4]. The burden of GERD is also increasing globally [5], contributing to the increases in BAR incidence, and consequently EAC.

The esophageal microbiota has been proposed as a contributor to the progression towards BAR [6, 7]; however, the evidence remains relatively scarce, inconsistent [8, 9], and more supportive of a role for specific pathobionts rather than global composition shifts [10, 11]. The limited number of studies investigating the human esophageal microbiome has by large focused on changes in prevalence or relative abundances of microbial taxa, with only one study by our group looking at changes in microbial function [10]. While these studies have provided insights into possible microbial agents of esophageal disease, they have lacked validation and have not causally linked the microbial changes with changes in the local host response, and there have not been attempts at delineating disease-promoting capabilities of microbial signatures of interest.

Given the similarities between the etiology of EAC and gastric adenocarcinoma [11], what is anticipated is that if microbial species are involved in disease progression then they are likely involved in the early stages of the pathological cascade (i.e., GERD and metaplasia). Multi-omics strategies and integrative analyses have been utilized effectively to identify factors central to disease etiology [12]. Thus, we employed a multi-omics strategy to profile the esophageal microenvironment in GERD and BAR, identifying host and microbial signatures putatively associated with disease progression in the esophagus. The associated bacterial species were then isolated from the same patients through targeted culture strategies, and novel genomic features associated with increased intracellular fitness in primary immune cells were identified. Screening of these microbial genomic features in shotgun metagenomics data of another cohort revealed a differential prevalence across the early stages of the EAC cascade. Co-culture of bacterial isolates with primary esophageal epithelial cells identified transcriptomic changes unique to those of relevance to disease.

## Methods

### Recruitment of subjects and nucleic acid extraction

To assess the esophageal microenvironment in GERD and the development of metaplasia, 48 subjects who underwent upper gastrointestinal endoscopy at the Prince of Wales Hospital (Sydney) for examination of their gastrointestinal symptoms were recruited prospectively (Table 1). Subjects who had a normal esophagus by histological assessment were considered controls. Subjects who had been prescribed antibiotics or non-steroidal anti-inflammatory drugs in the 2-month period prior to recruitment as well as subjects with other esophageal disease (squamous cell carcinoma, *n* = 1) were excluded. Two research samples, an esophageal mucosal biopsy and a saliva sample, were collected at endoscopy in addition to the required clinical samples. Researchers were blinded to the results of the histological analysis until sequencing was completed. Patients with GERD (*n* = 13) were uniformly graded by one endoscopist according to the Los Angeles classification [13] and were classified into the grades A (*n* = 9) and B (*n* = 4). No other findings at endoscopy were reported in any of the patients recruited. Nucleic acids were extracted from saliva and esophageal mucosal biopsies using the AllPrep DNA/RNA Mini Kit (Qiagen). RNA was further purified using 8 M LiCl. In addition to the current prospectively recruited cohort, 16S rRNA amplicon and shotgun metagenomics sequencing data from a previously published cohort (Table 1) [10], recruited at the Prince of Wales Hospital (Sydney), were employed. Data from esophageal brushings of 100 subjects [normal (NORM), *n* = 59; GERD, *n* = 29; metaplasia (MET), *n* = 12] who underwent upper gastrointestinal endoscopy for examination of their gastrointestinal symptoms were assessed.

**Table 1:** Clinical diagnosis, symptoms, and information of subjects. Within the new prospective cohort, patients with tissue metaplasia (MET) were older than patients with normal esophagi (*P* = 0.032—ANOVA post hoc Tukey’s test). While mean BMI increased with the progression of the cascade, no significant differences were observed (ANOVA post hoc Tukey’s test). As the researchers were blinded to clinical diagnosis until after sequencing, one patient found to have squamous cell carcinoma was excluded from further analyses. Within the published cohort, no significant differences in age were identified across groups (F_2,97_ = 1.19, *P* = 0.31; ANOVA). Patient BMI was not available. *NORM* normal, *GERD* gastro-esophageal reflux disease, *MET* metaplasia, *M* male, *Y* yes, *PPI* proton pump inhibitor, *BMI* body mass index. Age and BMI ± standard error of the mean

Cohort	Disease	Number (%)	Age (years)	Gender (M)	BMI	Reflux symptoms (Y)	PPI (Y)
Prospective	NORM	27 (57.4)	51.2 ± 3.1	7	25.4 ± 0.7	8	13
	GERD	13 (27.6)	57.3 ± 4.9	5	26.3 ± 1.2	6	6
	MET	7 (14.9)	68.8 ± 4.0	4	28.6 ± 1.5	2	4
Published	NORM	59 (59.0)	53.1 ± 1.9	21	–	1	32
	GERD	29 (29.0)	52.0 ± 2.5	8	–	28	9
	MET	12 (12.0)	59.2 ± 3.6	10	–	9	9

### Host transcriptomics

To investigate the host transcriptome profile during progression to metaplasia, RNA was prepared for sequencing using the SMARTer Stranded Total RNA Pico preparation kit and sequenced using NovaSeq 6000 chemistry (S2 100bp paired-end run). The tool Salmon which uses a quasi-mapping approach was employed for quantifying transcript abundance from RNA-seq reads [14]. Cell type analysis was performed using CIBERSORTx [15]. Multivariate statistics were applied as described above for the microbiota data. The R package DESeq2 was used to identify differentially expressed genes across specific comparisons [16]. Pathway and disease analyses were performed using Enrichr [17].

Three tools were used for identification of all major types of alternative splicing events (Exon skipping, intron retention, A5SS, A3SS, and mutually exclusive exons). These included rMATS [18], Whippet [19], and PSI-Sigma [20]. In-house scripts [21] were developed in python to identify splicing events which led to possible functional switches. These included biotype changes (e.g., from transcripts coding for functional proteins in one condition to the transcripts leading to proteins marked for nonsense-mediated decay or processed transcripts without a protein product in the other condition) or the events which lead to changes in protein product due to frame-shift, thus, resulting in complete or partial loss of functional domains. The Bioconductor/R packages maser [22] and drawProteins [23] were employed for visualizations of the alterative splicing events in transcripts in context of their protein products. PANTHER was employed for gene ontology enrichment analysis [24]. The tool CIRCexplorer2 [25] was used for identification and characterization of circular RNAs. Fusion genes were identified using the tool STAR-Fusion [26]. This tool uses the STAR aligner to map Illumina short reads, and then proceeds to use the junction as well as the spanning reads from the mapping output to the reference annotation to identify gene fusions.

### 16S rRNA amplicon sequencing

To examine the differences between the salivary (S) and esophageal (E) microbiotas, as well as determine any changes with disease progression, the full-length 16S rRNA gene was amplified using a KAPA HiFi HotStart PCR Kit and the primers 27F (/5AmMC6/ gcagtcgaacatgtagctgactcaggtcac AGRGTTYGATYMTGGCTCAG) and 1492R (/5AmMC6/ tggatcacttgtgcaagcatcacatcgtag RGYTACCTTGTTACGACTT) and sequenced on a PacBio RSII platform. The cycling conditions were 95 °C for 3 min, followed by 34 cycles for biopsies or 27 cycles for saliva, of 95 °C for 30 s, 57 °C for 30 s, and 72 °C for 60 s. Raw reads were analyzed using Mothur v1.39.1 [27, 28]. The resultant operational taxonomic unit (OTU) count matrix was used for statistical analysis (mean read depth: 1893 ± 101 clean reads/sample). OTUs generated from this data were termed pOTUs.

The V4 region of the 16S rRNA gene was also amplified using the earth microbiome primers (515F-806R), and sequencing was performed with Illumina MiSeq 2 × 250 bp chemistry at the Ramaciotti Centre for Genomics, UNSW Sydney, as previously described [10]. Raw reads were analyzed using Mothur v1.44.0 and vsearch v2.13.3 [27, 28]. E24 did not sequence to saturation and was removed along with its matching saliva sample (S24) from downstream analyses. The subsampled OTU count matrix was used for analysis (read depth: 13898 clean reads/sample). OTUs generated from this analysis were termed iOTUs. The prospective and published cohorts (Table 1) were then combined, and raw reads again analyzed using Mothur v1.44.0 and vsearch v2.13.3. The subsampled OTU count matrix was used for analysis (read depth: 13,898 clean reads/sample). OTUs generated from this analysis were termed ciOTUs.

α-diversity measures, Euclidean distances, Bray-Curtis resemblances, principal coordinate analysis (PCoA), distance-based redundancy analysis (dbRDA), ANOSIM, and distance-based linear models (permutational multivariate ANOVA) were calculated using Primer-E v6 (Quest Research Limited; Auckland, New Zealand). The models included the variables: location (saliva or esophageal), subject age, sex, proton pump inhibitor (PPI) use, body mass index (BMI), reflux symptoms, and disease (NORM, GERD, MET) and were tested against Euclidean distances for α-diversity measures and Bray-Curtis resemblances for beta-diversity. Per taxon analyses were performed using LEfSe [29]. Source tracking of esophageal taxa against saliva samples was performed using SourceTracker [30], within the Metagenomics for Environmental Microbiology Galaxy framework [31]. All other tests were performed using GraphPad Prism v8 (GraphPad Software; San Diego, CA, USA).

### Associations between microbiome and host transcriptome

To determine if any associations exist between the host transcriptome and microbiota profiles, correlations of resemblance matrices were performed using the RELATE function in Primer-E v6. Procrustes and protest analyses were performed using the R package “vegan.” Tests were performed on both PCoA and dbRDA axes calculated for Bray-Curtis resemblance matrices of microbiome (transformed relative abundances) and host transcriptome data (normalized counts). Non-parametric correlations were calculated using the framework outlined in Reshef et al. [32] and accessible through the R package “minerva.” Inputs were transformed relative abundances of *Campylobacter* taxa and normalized transcript counts.

### Isolation of motile bacterial species from the patient cohort

To culture members of the microbiota of relevance to disease progression, patients’ saliva and esophageal mucosal biopsy samples were resuspended in 1× phosphate buffered saline (PBS), homogenized, and cultured at 37 °C for 48 h on Horse Blood Agar (HBA) [Blood Agar Base No. 2 (Oxoid, Melbourne, Vic, AU) with 6% defibrinated horse blood (Oxoid)] containing 10 μg/ml vancomycin (Sigma-Aldrich; Sydney, NSW, AU) under anaerobic conditions with hydrogen enrichment [10% hydrogen, 5% CO_2_, ~ 0.5% O_2_]. These conditions were generated using an AnaeroGen gas pack (Oxoid) and 0.073 g sodium borohydride (Sigma-Aldrich). The resultant culture was filtered through a 0.6 μM membrane (Millipore, Melbourne, Vic, AU), and the filtrate subcultured on HBA under the same conditions. Single colonies were selected from each culture’s filtrate and were harvested into Brain Heart Infusion broth and glycerol (70:30 v/v). To confirm isolates were *Campylobacter* species, the 16S ribosomal RNA gene was amplified directly from colonies through *Campylobacter*-specific PCR using the C412F and C1288R primer pair (40 cycles of 95 °C for 10 s, 55 °C for 10 s, and 72 °C for 45 s). For additional validation, PCR products were Sanger sequenced using BigDye chemistry and their identity confirmed using BLASTn searches against the NCBI database. Positive isolates were stored at − 80 °C for further use.

### Culture of primary macrophages and infection with bacterial isolates

A range of *Campylobacter* species are known to invade and survive within epithelial cells; however, there is no consistent association between their capacity to do so and the disease status of the host from which they were isolated from. We postulated that isolates that induce more amplified responses from immune cells or that could overcome their defenses would more likely correspond to persistent pathobionts that have a chronic effect on the host.

Primary human monocyte-derived macrophages were prepared from buffy coats (100 ml) obtained from eight healthy donors through the Australian Blood Services (Australian Red Cross material supply agreement: 18-01NSW-06) under strict LPS-minimized conditions as described [33]. In brief, PBMCs were isolated using density gradient centrifugation (Ficoll-Paque Plus; Amersham Biosciences). PBMCs washed twice with PBS were suspended at ∼1 × 107 cells/ml in RPMI 1640 containing 2 mM l-glutamine, 10 U/ml penicillin and 100 mg/ml streptomycin, and 10% heat-inactivated Ab serum (Sigma). Cells were incubated at 37 °C and 5% CO_2_ in 24-well Corning Costar plates and non-adherent cells removed after 1.5–2 h yielding 2–3 × 10^6^ monocytes/ml/well (> 90% as confirmed by CD14 staining). Cells were then cultured in RPMI 1640 complete media containing 10% AB serum supplemented with 20 ng/ml M-CSF (BioSource) for 3 days, then washed twice with PBS and cultured for another 4 days in culture medium without M-CSF. Primary macrophages were infected with each of the different *Campylobacter* isolates from our cohort at a MOI of 100, supernatants were collected (4 and 18 h) for multiplex ELISA, and gentamicin protection assays were performed as previously described [34]. Then, 1 μM Latrunculin A (Sigma) was added to a subset of experiments to block phagocytosis. The acidification of lysosomes upon infection was tracked using 2.5% LysoTracker Green DND-26 (Thermo Fisher; North Ryde, NSW, AU) as outlined by the manufacturer.

### Multiplex ELISA

To establish the inflammatory response of healthy primary macrophages to patient *Campylobacter* isolates, 34 cytokines and chemokines were measured in the co-culture supernatants using the Cytokine & Chemokine 34-Plex Human ProcartaPlex™ Panel 1A (Jomar Life Research; Scoresby, VIC, Australia) according to the manufacturer’s instructions. A Luminex MAGPIX instrument with xPONENT software (Luminex Corporation; Northbrook, IL, USA) was calibrated with MAGPIX Calibration and Performance Verification Kits (EMD Millipore; Billerica, MA, USA) and employed to acquire data. Data was analyzed with Multiplex Analyst software version 5.1 (Luminex) as the Median Fluorescent Intensity using spline curve-fitting for calculating analyte concentrations.

### Genome sequencing of isolates and comparative genomics

To identify genetic features that differentiate the *Campylobacter* isolates, bacteria were grown as described and DNA was extracted using the Isolate II Genomic DNA Kit (Bioline; Cat number: BIO-52066). DNA from each isolate was prepared for sequencing on individual PacBio RSII sequencing cells using the 10–20 kb Genomic RSII library preparation kit. Bacterial DNA was also prepared using Nextera XT DNA library preparation kit and sequenced using MiSeq v3 2 × 300 bp chemistry at the Ramaciotti Centre for Genomics. The package SolexaQA [35] was used to calculate sequence quality statistics and trim the input fastq files by quality. The analysis included quality trimming of the reads to the user-supplied quality cutoffs using the module dynamictrim. This was followed by trimming the reads by user-defined length cutoff using the module lengthsort. The assembly of individual bacterial strains was performed using the Canu assembler [36], specialized for noisy single-molecule sequences. The tool Pilon [37] was then employed to refine the draft assembly by correcting bases, fixing mis-assemblies, and filling gaps with the help of high accuracy Illumina short-read datasets from the same samples. The final refined genome assemblies were annotated using the highly accurate and fast command line tool Prokka [38]. Average nucleotide identity (ANI) was calculated using OrthoANI [39] and genomes were visualized using GView [40]. Comparative genomics to identify strain-specific orthologous proteins was performed using the tool Proteinortho [41].

### Assessment of resistance to lysozyme

The susceptibility of isolates to lysozyme was assessed either by exposure to 1 mg/ml human lysozyme (Sigma) or a combination of 0.3 mg/ml lysozyme and 3 mg/ml human lactoferrin (Sigma) for 6 h.

### Generation of bacterial mutants deficient in LprI_01601 and LprI_00928

The *C. concisus* ΔLprI_01601 and *C. rectus* ΔLprI_00928 mutants were generated using an adapted protocol involving allelic exchange [42]. A linear DNA template was designed to contain fragments of about 500 bp upstream and downstream of the target gene flanking a kanamycin resistance cassette from *Campylobacter jejuni* (accession number: M29953.1). Both linear templates, incorporated into a pET-3a plasmid within the BamHI site, were generated by GenScript (Piscataway, NJ, USA). The *C. concisus* template was amplified from the plasmid by flanking primers (CC1601A: ACGAGGCTAGCGTTTTTAGC; CC1601D: GCTAATAGTTTTCAAGCTGCATTC), purified, methylated using 200 μg of *C. concisus* ESOS44-1 lysates with 0.4 mM S-Adenosylmethionine (Sigma) as previously outlined [43], and naturally transformed into *C. concisus* ESOS44-1 grown for 4 h. The *C. rectus* template was amplified from the plasmid by flanking primers (CR00928F1: TCCACAGAAAAGCTCATATCC; CR00928R1: TTCGTACTCGCAGCTCGTCAA), purified, methylated using 200 μg of *C. rectus* ESOS44-4 lysates as above, and naturally transformed into *C. rectus* ESOS44-4 grown for 4 h. Isolates were transferred onto kanamycin-containing HBA plates (20 μg/ml) after 18 h. The *C. concisus* mutant was validated through PCR of the target gene using various combinations of the flanking primers with primers internal to LprI_01601 (1601F: GTAAGCAATACACAAGATATTGAAG; 1601R: TTGCTAACTCTTCCGCTCTT). The *C. rectus* mutant was validated using amplification with the above primers (> 1700 bp fragment) and Sanger sequencing with BigDye Terminator v3.1 chemistry. Of note, transformation efficiency of *C. rectus* was lower as the bacterium had a higher tolerance to kanamycin, and thus, an increased concentration for selection may be more suitable.

### Expression of recombinant LprI_01601 and immunoprecipitation

The sequence of LprI_01601 from ESOS44-1 with a C-terminal His-tag was inserted into a pET-30a(+) plasmid between NdeI and HindIII, expressed in *E. coli*, and purified by Ni column. Plasmid sequences were validated by Sanger sequencing, and protein expression purification was validated by SDS-PAGE and anti-His-Tag Western blot analysis. All services were performed by GenScript. Interaction partners were then identified by using LprI_01601 as a bait and primary macrophage non-detergent cell lysates as target co-immunoprecipitated with magnetic bead conjugated anti-His-tag monoclonal antibody as previously described [44]. The experiment was repeated twice, and all fractions were analyzed using LC/MS-MS [45]. Only proteins identified in both repeats with a mascot score > 60 in at least one experiment and not identified in either of the negative controls (anti-His-tag IP without LprI_01601) were retained. The list was then further filtered for keratins, immunoglobulins, histones, and ribosomal proteins.

### Co-culture of primary esophageal epithelial cells with *Campylobacter* isolates

Human primary esophageal epithelial cells (Cell Biologics; H-6046) were grown on the recommended Complete Human Epithelial Cell Medium (Cell Biologics; H-6621) in coated 24-well tissue culture plates at a concentration of 1.5 × 10^4^ cells/ml. The cell culture media was replaced with antibiotic-free media then representative *Campylobacter* isolates (ESOS13-1, ESOS18-1, ESOS14-1, ESOS33-1, ESOS44-1, and ESOS44-4) grown as described above, were added to the human cells at a MOI of 10 for 4 h. RNA was extracted using the RNeasy Plus Mini Kit (Qiagen) and prepared using the Stranded mRNA library kit (Illumina). Libraries were then sequenced on the NovaSeq 6000 system (flow cell type: SP; chemistry: 1 × 100 bp) producing a mean 21,735,733 ± 311,229 clean reads across all samples. Data were analyzed as above, utilizing Salmon [14] and DESeq2 [16] to identify differentially expressed genes, as well as Enrichr [17] for pathway analyses.

## Results

### Profound changes in the host esophageal transcriptome occur in metaplasia but not GERD

**Fig. 1:**
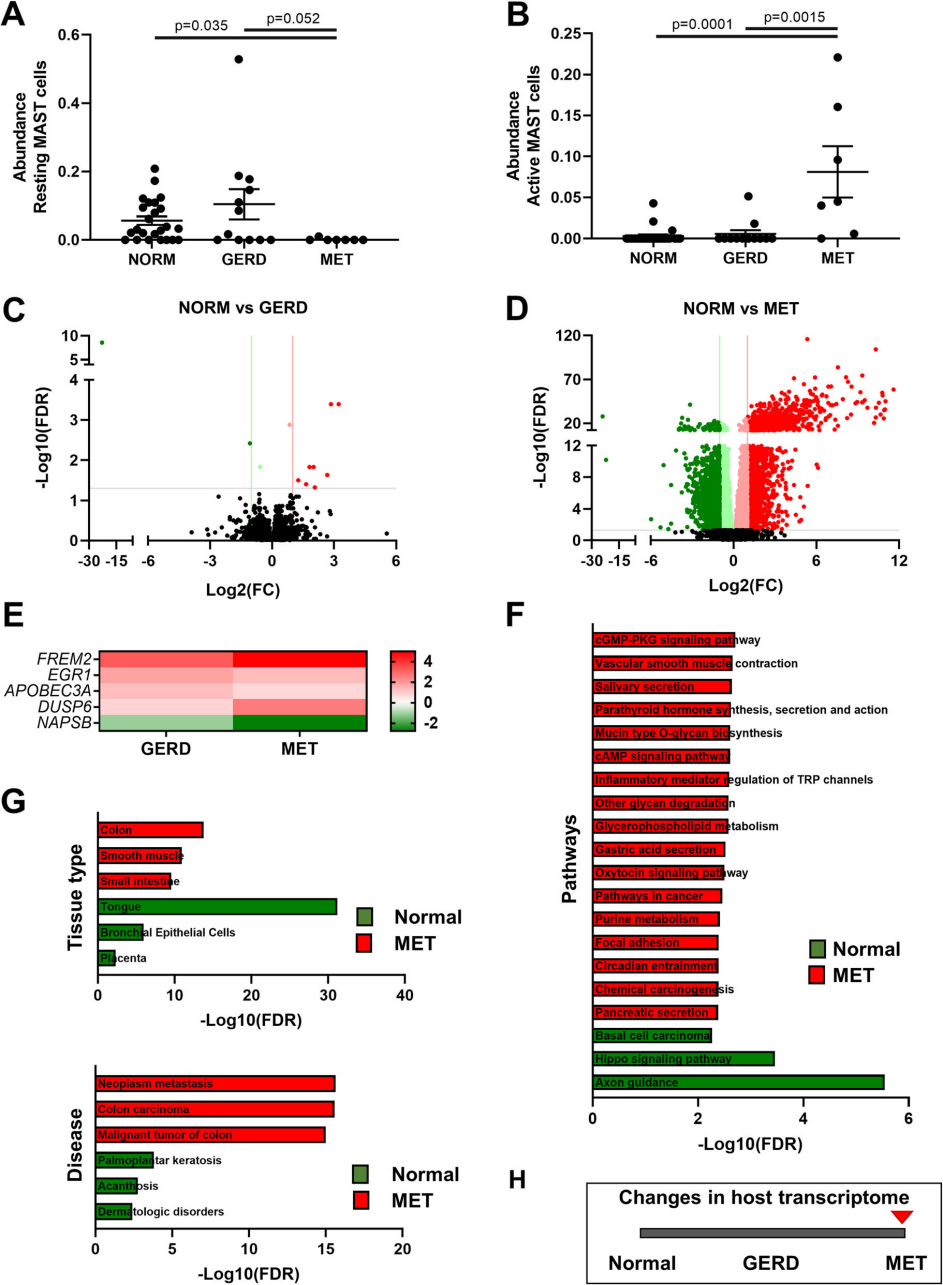
Host transcriptome changes in the esophagus of patients with GERD and metaplasia. Shotgun sequencing of ribosomal-depleted RNA was performed on a NovaSeq platform. **A** Changes in predicted abundance of resting mast cells across the different groups. **B** Changes in predicted abundance of active mast cells across the different groups. Abundances were calculated using CIBERSORTx and statistical differences were tested using Kruskal-Wallis with Dunn’s multiple comparisons test. **C** Volcano plot of differentially expressed genes between NORM and GERD samples. **D** Volcano plot of differentially expressed genes between NORM and MET samples. Differential expression was calculated using DESeq2. Red, upregulated in disease state, Green, downregulated in disease state. **E** Genes consistently regulated across GERD and MET when compared to NORM samples. Scale bar is fold-change. **F** Pathway analysis of differentially expressed genes in MET samples. **G** Tissue type and disease analyses of differentially expressed genes in MET samples. All analyses were conducted using Enrichr. **H** Significant changes in the host esophageal transcriptome occur at the time of metaplasia

To examine the changes in the host transcriptional profile in GERD and MET, bulk shotgun sequencing of RNA from biopsy samples from prospectively recruited subjects (Table 1) was performed. The immune cell profile within the biopsies was estimated using CIBERSORTx, and analyzed against all variables (age, sex, PPI use, BMI, reflux symptoms, and disease) with a distance-based linear model. No significant differences were identified in the cell profiles. However, a borderline non-significant difference was found for disease (Pseudo-F: 2.0, *P* = 0.057, df = 41) that reached significance on one-way analysis against disease (*R* = 0.152, *P* = 0.035; ANOSIM) and was driven by differences between MET and the other groups (NORM: *R* = 0.291, *P* = 0.018; GERD: *R* = 0.214, *P* = 0.023). Specifically, lower levels of resting mast cells and higher levels of active mast cells were found in MET samples when compared to NORM and GERD (Fig. 1A, B).

Changes in host transcription profiles were then assessed using a distance-based linear model on log-transformed counts (Bray-Curtis resemblance) accounting for same variables as above, with the strongest differences observed for disease (Pseudo-F: 11.6, *P* = 0.001, df = 41), followed by BMI (Pseudo-F: 3.1, *P* = 0.012, df = 41), age (Pseudo-F: 2.8, *P* = 0.016, df = 41), and sex (Pseudo-F: 2.1, *P* = 0.033, df = 41). The changes in transcript levels in disease were driven once again by differences between MET samples and the other groups (NORM: *t* = 4.05, *P* = 0.001, df = 29; GERD: *t* = 2.94, *P* = 0.001, df = 17, PERMANOVA), with no difference observed when NORM and GERD were compared (*t* = 1.12, *P* = 0.14, df = 34, PERMANOVA). Differentially expressed transcripts were then identified, with only 12 significantly regulated between NORM and GERD (Fig. 1C; Additional file 1: Table S1) as compared to 7884 between NORM and MET (Fig. 1D, Additional file 1: Table S2), confirming the multivariate analyses. Notably, of the 12 differentially expressed transcripts in GERD, 5 were differentially expressed in the same direction in MET (Fig. 1E), showing some consistency across the two stages.

Pathway analysis of differentially regulated transcripts in MET was then performed, with key pathways known to be altered in BAR identified as enriched. These pathways included mucin glycan biosynthesis, oxytocin signaling, pathways related to carcinogenesis, and those related to acid and bile secretion (Fig. 1F; Additional file 1: Table S3). Enrichment of Fc gamma R-mediated phagocytosis and Lysosome-related proteins was also observed (Additional file 1: Table S3). Tissue type and disease enrichment analyses confirmed expression in MET resembled that in the colon, and specifically, colonic malignancy (Fig. 1G). FOXA2, Cathepsin E (CTSE), REG4, TFF1, TFF2, the bicarbonate channel CFTR, and PDZ-domain-containing mucins (MUC17, MUC3A, and MUC12) were among the most highly upregulated transcripts in MET.

In addition to cell types and expression profiles, changes in splicing, circular RNAs, and fusion events were also interrogated. A substantial number of putative splicing events were identified in both GERD and MET when compared to NORM samples (Additional file 1: Table S4), with a strict assessment of exon skipping and intron retention events highlighting that a number of these events can potentially lead to changes in protein amino acid sequence due to nonsense-mediated decay (Additional file 2: Figures S1, S2; Additional file 1: Table S5). Enrichment analysis showed that differentially spliced transcripts in GERD were populated by transcripts involved in the cellular response to stress (fold enrichment = 3.37, *P* = 4.25 × 10^−9^, FDR = 6.75 × 10^−5^), whereas those in MET were populated by transcripts involved in stress granule formation (fold enrichment = 29.79, *P* = 1.75 × 10^−6^, FDR = 0.0278).

Differences in the prevalence of circular RNAs (Additional file 2: Figure S3A; Additional file 1: Table S6) and fusion events (Additional file 2: Figure S3B; Additional file 1: Table S7) across the three groups were also identified, in the majority of cases MET being different to NORM and GERD. Two notable exceptions were the circular RNAs *CUL6*, showing consistent prevalence in GERD and MET, as well as *GOLM1* showing a stepwise increase in disease (Additional file 2: Figure S3A).

The above findings indicate that while some changes in host profile can be observed in GERD, most changes occur at the stage of or during progression to MET (Fig. 1H).

### The esophageal microbiota is distinct from the salivary microbiota with *Campylobacter* being enriched in reflux and metaplasia

**Fig. 2:**
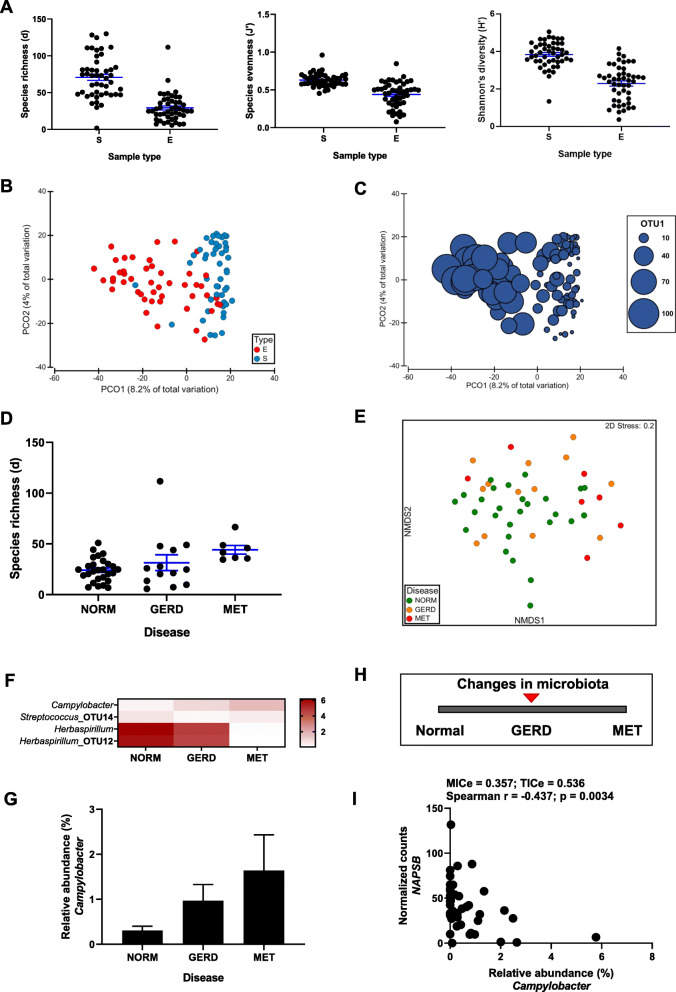
The saliva and esophageal microbiota in subjects with GERD and metaplasia. Full-length 16S rRNA amplicon sequencing was performed on a PacBio platform. **A** Comparison of alpha diversity measures between saliva (S) and esophageal (E) samples. Significant differences between sample types (Richness: Pseudo-F: 69.7, *P* = 0.001, df = 92; Evenness: Pseudo-F: 48.7, *P* = 0.001, df = 92; Shannon’s diversity: Pseudo-F: 83.2, *P* = 0.001, df = 92) were observed across all measures using linear models that corrected for age, sex, PPI, BMI, reflux symptoms, and disease. **B** Principal coordinate analysis of Bray-Curtis resemblance matrix generated from square-root-transformed pOTU relative abundances. Distance-based linear models corrected for all variables identified significant differences in composition between sample types (Pseudo-F: 5.1, *P* = 0.001, df = 92). **C** The same principal coordinate analysis as above incorporating the relative abundance of *Streptococcus* pOTU1. **D** Esophageal species richness stratified according to disease. **E** Non-metric multidimensional scaling plot of Bray-Curtis resemblance matrix generated from square-root-transformed OTU relative abundances. Only esophageal samples were plotted. **F** Heatmap of genera and OTUs identified by LEfSe to be significantly different (LDA > 3) between normal and GERD (Additional file 1: Table S9) or normal and MET (Additional file 1: Table S10). **G** Mean relative abundance of *Campylobacter* stratified according to disease. Errors are SEM. **H** Significant changes in the esophageal microbiota occur by the time of diagnosis of GERD. **I** A significant co-exclusion relationship between the relative abundance of *Campylobacter* and *NAPSB* was present. Non-parametric correlations were identified through MINe and confirmed with Spearman’s correlation

To establish that the esophageal microbiota is a distinct microbial community, matched saliva and esophageal samples from the same recruited subjects (Table 1) were first profiled using full-length 16S RNA amplicon sequencing (PacBio) and community metrics were compared. The esophageal microbiota had significantly lower alpha diversity measures (Fig. 2A) and significantly different composition (Fig. 2B), even after correction for subject age, sex, PPI use, BMI, reflux symptoms, and disease. Many taxa were significantly different in relative abundance across sample types (Additional file 1: Table S8), the taxon showing the largest differential relative abundance being *Streptococcus* pOTU1 (LDA = 5.16, *P* < 0.0001; Fig. 2C). Despite the differences between communities, as expected, source tracking of microbial counts indicated that most of the esophageal taxa originated from the oral cavity (mean ± STD: 0.84 ± 0.27; 95% CI 0.76–0.92).

Given that the two communities were distinct, their associations with different predictors were analyzed independently. In a model that included subject age, sex, PPI use, BMI, reflux symptoms, and disease, a significant association was identified between disease and esophageal species richness (Pseudo-F: 7.4, *P* = 0.005, df = 45), marked by an increase in richness with disease progression (Fig. 2D). Models incorporating the same variables also identified a significant shift in esophageal microbiota composition (Pseudo-F: 1.4, *P* = 0.02, df = 45; Fig. 2E), which was confirmed on one-way analysis (*R* = 0.178, *P* = 0.008; ANOSIM). To identify the taxa responsible for this shift, per taxon analyses were performed using LEfSe (Additional file 1: Tables S9-11), and a striking enrichment of *Campylobacter* was observed in disease (Fig. 2F,G; GERD: LDA = 3.53, *P* = 0.018; DIS: LDA = 3.66, *P* = 0.012). No associations were observed with the saliva microbiota, except for a trend towards a shift in composition with BMI (Pseudo-F: 1.1, *P* = 0.063, df = 45).

To confirm the observed outcomes using a different platform, the V4 region of the 16S rRNA gene was sequenced (Illumina), and the same tests applied. Differences were once again observed between the saliva and esophageal communities, with lower alpha diversity measures in the esophagus (Additional file 2: Figure S4A), a compositional shift (Additional file 2: Figure S4B), and higher relative abundance of *Streptococcus* iOTU2 (LDA = 4.98, *P* < 0.0001; Additional file 2: Figure S4C; Additional file 1: Table S8). Consistently, a trend towards increased esophageal species richness in disease was observed (Pseudo-F: 4.3, *P* = 0.052, df = 44; Additional file 2: Figure S4D), in addition to an association between esophageal species richness with BMI (Pseudo-F: 3.5, *P* = 0.048, df = 44). Differences in esophageal microbiota composition with disease had a similar effect size to above but did not reach significance (Pseudo-F: 1.37, *P* = 0.12, df = 44). Notably however, when patients with GERD and Barrett’s were combined, the associations between richness and disease (Pseudo-F: 6.3, *P* = 0.018, df = 44) as well as composition and disease (Pseudo-F: 1.6, *P* = 0.038, df = 44) reached significance, suggesting that the resolution from profiling a smaller fragment may contribute to the statistical power to identify differences. The association between the saliva microbiota composition and BMI was confirmed using this platform (Pseudo-F: 2.1, *P* = 0.017, df = 44). To validate the taxa driving these differences, LEfSe analysis were performed (Additional file 1: Tables S9-11), and a taxon classified to *Campylobacter* was found to be enriched in disease (GERD: LDA = 3.28, *P* = 0.0021; DIS: LDA = 3.17, *P* = 0.0029; Additional file 2: Figure S4E,F). This taxon (iOTU36) was also more prevalent in disease [NORM: 26.9% (7/26); GERD: 76.9% (10/13); MET: 57.1% (4/7); *χ*^2^ = 9.17, *P* = 0.010].

To validate the relevance of *Campylobacter* in a larger cohort, the current cohort was re-analyzed in combination with a previously published cohort of esophageal brushings from subjects with normal esophagi, GERD, or metaplasia [Table 1 [10];]. While a strong batch effect was detected (Additional file 2: Figure S5A), the same *Campylobacter* taxon (cOTU65; different OTU assignment but same consensus sequence) was consistently found to be enriched in disease (GERD: LDA = 3.04, *P* = 0.0018; MET: LDA = 2.87, *P* = 0.021; DIS: LDA = 3.02, *P* = 0.00048; Additional file 2: Figure S5B,C; Additional file 1: Tables S9-11).

The findings indicate that the esophageal microbiota is distinct, and changes in richness and composition occur at GERD (Fig. 2H) and are driven in part by consistent enrichment of specific taxa such as *Campylobacter*.

### *Campylobacter* levels were correlated with inferred levels of active mast cells and expression of a lysosomal transcript

The relationship between the esophageal microbiome and host transcriptome in this cohort was assessed to determine if any associations exist. Significant concordance between the microbiome (PacBio) and transcriptome data sets (PCoA: m_12_ = 0.454, *P* = 0.001; dbRDA: m_12_ = 0.206, *P* = 0.001) were observed (Additional file 2: Figure S6A). The matrices were also found to be significantly correlated (Spearman Rho: 0.309, *P* = 0.004). A similar significant concordance was seen between the transcriptome and the microbiome (Illumina) data sets (PCoA: m_12_ = 0.264, *P* = 0.001; dbRDA: m_12_ = 0.175, *P* = 0.001) (Additional file 2: Figure S6B) but the correlation did not reach significance (Spearman Rho: 0.083, *P* = 0.19). To confirm the validity of the relationships, a random matrix was generated, and no significant correlation was observed against the transcriptome data set (Spearman Rho: − 0.041, *P* = 0.727).

Next, the associations between the strongest microbial signature in our data (relative abundance of *Campylobacter*) and the host transcriptome were assessed. A strong correlation between the relative abundance of *Campylobacter* (PacBio) and the abundance of active MAST cells was observed (Pearson R = 0.703, 95% CI 0.510–0.828, *P* = 0.0001, FDR = 0.002). Non-parametric correlations between transcript counts and the relative abundances of *Campylobacter* (PacBio, Additional file 1: Table S12) and *Campylobacter* iOTU36 (Additional file 2: Figure S7A; Additional file 1: Table S13) were also calculated, and a range of exponential relationships with molecules were identified (Additional file 2: Figure S7B). Notably, a significant co-exclusion relationship between the relative abundances of *Campylobacter* taxa and *NAPSB* were present (both of our data sets, Fig. 2I; Additional file 2: Figure S7C), this pseudogene being found to be consistently significantly downregulated in GERD and MET when compared to NORM samples (Fig. 1E).

### Targeted culture identifies *Campylobacter* isolates with increased capacity to survive in primary macrophages

In parallel to the sequencing strategies, several culture strategies for the isolation of microaerobic and anaerobic bacteria were implemented on the same saliva and esophageal mucosal samples, including a filter strategy [34] for the isolation of *Campylobacter* species. Eleven *Campylobacter* isolates were grown from saliva samples of normal (*n* = 3), GERD (*n* = 4), and MET (*n* = 4), 10 of which were putatively identified as *Campylobacter concisus* and 1 as *Campylobacter rectus* on 16S rRNA amplicon sequencing with Sanger sequencing.

Despite the fact that the salivary and esophageal microbiotas were found to be distinct in profile in this cohort, our source tracking analysis revealed that the majority of the detected esophageal bacteria did originate from the oral cavity. This would indicate that strains isolated from the patient’s saliva were good representatives of those found in their esophagus. Thus, the pro-inflammatory potential of these isolates was assessed through co-culture with primary human macrophages and measurement of the levels of 34 cytokines and chemokines at two time points (4 and 18 h). The capacity of these isolates to survive within primary macrophages was also determined at 4 h, given that a co-exclusion relationship between *Campylobacter* and lysosomal transcripts was identified.

**Fig. 3:**
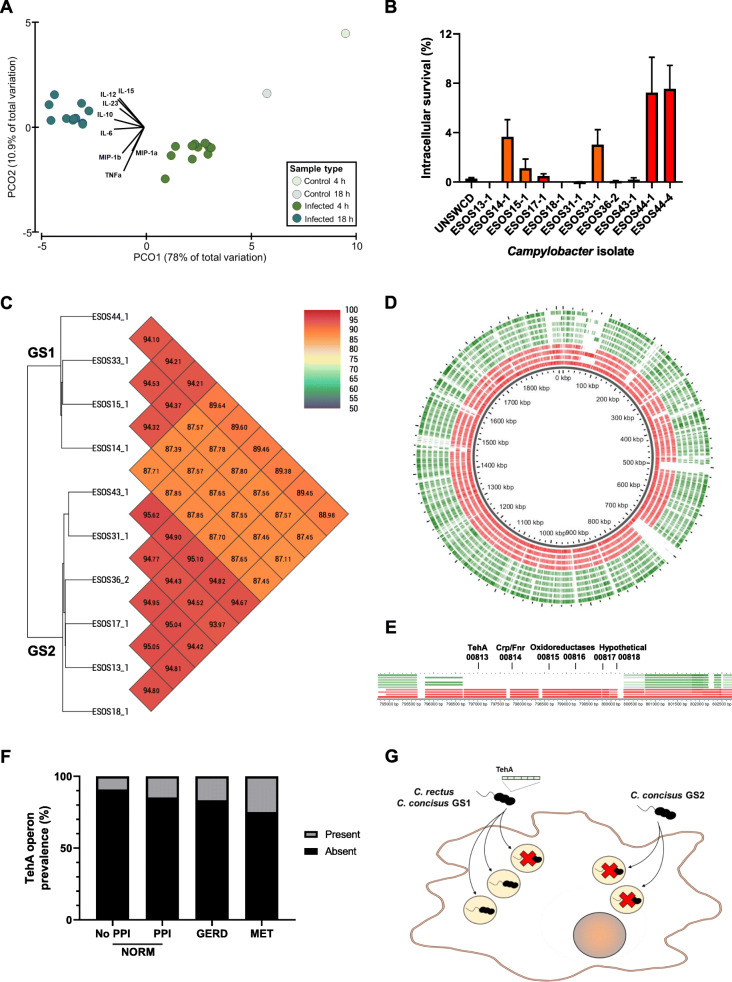
Virulence and genomic differences across *Campylobacter* isolates from patients with GERD and metaplasia. **A** Principal coordinate analysis of the levels of 34 cytokines and chemokines produced by primary macrophages upon infection with *Campylobacter* isolates. Macrophages were infected with 10 different *C. concisus* and 1 *C. rectus* isolate from this patient cohort. Data was log(*x* + 1) transformed and a Euclidean distance matrix generated. Vectors were generated using a correlation value of 0.3 for both Pearson and Spearman correlations. **B** Intracellular levels of *Campylobacter* isolates within primary human macrophages. Levels were calculated using a gentamicin protection assay at MOI 100. ESOS44-1 and ESOS44-4 were significantly different to all other isolates except for ESOS14-1, ESOS33-1, and each other (ANOVA, Tukey’s). No other comparisons were significant. Red: isolates from MET, orange: from GERD, and green: from NORM. **C** Average nucleotide identity across the 10 *C. concisus* isolates’ genomes. A separate analysis including *C. rectus* showed an ANI of 71.02–71.39%. **D** Circular representation of the 10 *C. concisus* genomes using GView. GS1 strains, red; GS2 strains, green. **E** Syntenic proteins unique to GS1 strains as compared to GS2 strains, including the tellurite resistance protein TehA. **F** Prevalence of the TehA operon in shotgun metagenomics data of esophageal mucosal brushings from patients in the early stages of the EAC cascade. Reads aligning to the TehA operon within the data were identified using BWA-MEM. **G** *C. concisus* GS1 isolates and *C. rectus* have enhanced intracellular survival in primary macrophages when compared to *C. concisus* GS2 isolates, and the former can be delineated by the presence of six syntenic proteins including TehA

While significant differences in cytokine and chemokine production was observed between non-infected and infected cells (4 h: *t* = 4.36, *P* = 0.016, df = 11; 18 h: *t* = 8.01, *P* = 0.013, df = 11), and between 4 and 18 h infection time points (*t* = 8.56, *P* = 0.001, df = 20), no differences were found across disease groups (*p* > 0.67 for all) (Fig. 3A). In contrast, substantial differences in the capacity of the isolates to survive within primary macrophages were identified (Fig. 3B). Two isolates (*C. concisus* ESOS44-1 and *C. rectus* ESOS44-4) from a patient with MET and three isolates from patients with GERD (*C. concisus* ESOS14-1, ESOS15-1, and ESOS33-1) all had intracellular levels greater than 1%. No isolates from NORM showed high intracellular levels (0%, 0%, and 0.082%). Notably, a strain previously isolated from a patient with Crohn’s disease (UNSWCD), which had a strong capacity to invade intestinal epithelial cells [46], showed a weaker capacity (0.27%) to survive in primary macrophages.

### *C. concisus* genomospecies 1 has increased intracellular survival in primary macrophages and shares a set of six syntenic proteins with *C. rectus*

To identify the possible genetic basis for increased intracellular survival in macrophages by certain *Campylobacter* isolates, the genomes of all 11 strains were sequenced using both PacBio and Illumina chemistry. Unsupervised comparative genomics through average nucleotide identity (ANI) was applied on the 10 *C. concisus* genomes, and two unique clusters were identified, with isolates found to have increased survival (> 1%) clustering together (Fig. 3C). These clusters corresponded to the two *C. concisus* genomospecies (GS) previously identified [47], with GS1 being that which survives within primary macrophages.

Supervised comparative analyses identified 25 proteins unique to our four GS1 isolates when compared to the other six GS2 isolates, a substantial portion of which were involved in redox reactions, functions highly relevant in macrophage pathogen elimination machinery (Fig. 3D; Additional file 1: Table S14). Intriguingly, within these 25 proteins was a group of six proteins found conserved in synteny within the genome of the four GS1 isolates (Fig. 3E), including tellurite resistance protein TehA, a Crp/Fnr family transcriptional regulator, two oxidoreductases, and two DUF2892 domain-containing proteins. The whole syntenic group was also found to be highly conserved in *C. rectus* ESOS44-4 (average similarity 69–82%). Further, analyses of the presence of the 25 unique proteins across the 224 available *C. concisus* genomes showed that 88 strains had good conservation of these proteins, with the operon of interest containing TehA delineating this separation effectively (tblastn, Additional file 1: Table S14). This clustering was also concordant with clustering according to GS.

To test the prevalence of the TehA operon and TehA only within the esophageal microenvironment across the disease cascade, shotgun metagenomics data of esophageal brushings [10] were searched for reads matching these regions using BWA-MEM. A stepwise increase in the prevalence of reads mapping to these two regions was observed across the disease cascade (Fig. 3F; Additional file 2: Figure S8A); however, the increasing trend did not reach statistical significance (TehA operon: *χ*^2^ = 1.44, *P* = 0.23; TehA: *χ*^2^ = 1.53, *P* = 0.22; chi-square test for trend).

In summary, *C. concisus* GS1 isolates and *C. rectus* have enhanced intracellular survival in primary macrophages (Fig. 3G) and can be delineated by the presence of six syntenic proteins including TehA.

### *Campylobacter* isolates from a patient with metaplasia possess additional genetic elements that improve fitness

**Fig. 4:**
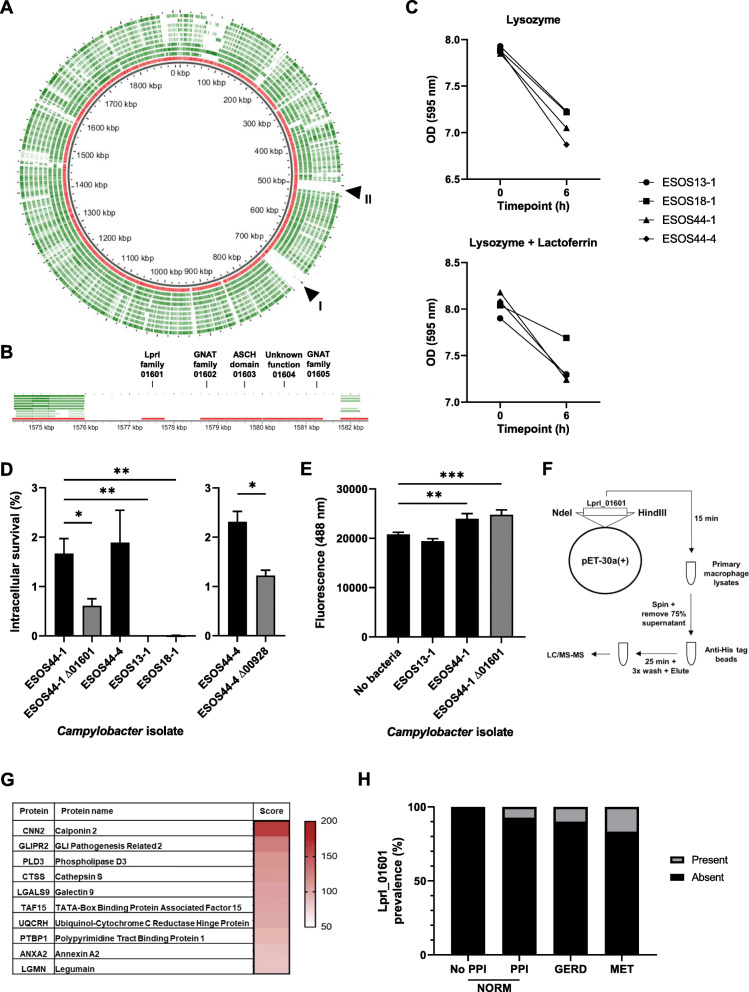
Characteristics of *C. concisus* ESOS44-1 and ESOS44-4 compared to other *C. concisus* isolates from this cohort. **A** Circular representation of the 10 *C. concisus* genomes using GView. ESOS44-1, red; other isolates, green. Region I: Prophage CP4-57; Region II: lipid A biosynthesis. **B** Syntenic proteins unique to ESOS44-1 as compared to other isolates, including LprI_01601. **C** Susceptibility of representative *Campylobacter* isolates to lysozyme as well as lysozyme and lactoferrin. Experiments were repeated in triplicate and representative results were presented. **D** Intracellular levels of representative wild-type *Campylobacter* isolates and the ΔLprI_01601 mutant within primary human macrophages. Levels were calculated using a gentamicin protection assay at MOI 100. ESOS44-1 was significantly different to all other strains except for ESOS44-4 (ANOVA, Dunnett’s; * *P* < 0.05, ** *P* < 0.01). Decreased intracellular levels of ESOS44-4 ΔLprI_00928 as compared to the wild-type isolate were validated in primary macrophages from another donor (Welch’s *t*-test; * *P* < 0.05). **E** Acidification of lysosomes following infection with wild-type *C. concisus* isolates and the mutant strain. Fluorescence was measured following the addition of LysoTracker Green. ESOS44-1 and the ΔLprI_01601 mutant were both significantly different to the negative control (ANOVA, Dunnett’s; ** *P* < 0.01, *** *P* < 0.001). **F** Experimental procedure for LprI_01601 recombinant expression and immunoprecipitation following incubation with primary macrophage lysates. **G** Human proteins that co-immunoprecipitated with LprI_01601. Proteins are arranged according to combined mascot score from two IP and LC/MS-MS experiments. **H** Prevalence of LprI_01601 in shotgun metagenomics data of esophageal mucosal brushings from patients in the early stages of the EAC cascade. Reads aligning to LprI_01601 within the data were identified using BWA-MEM

In addition to the increased intracellular survival of GS1 isolates, an even higher capacity to survive was noted for one isolate from a patient with BAR (ESOS44-1), and this increased capacity was shared with *C. rectus* ESOS44-4, isolated from the same patient (Fig. 3B). To identify genes unique to ESOS44-1 and shared by ESOS44-4, first, comparative genomic analyses were performed against the other nine *C. concisus* isolates, with a total of 60 proteins found to be unique to ESOS44-1 (Fig. 4A; Additional file 1: Table S15). A substantial number of these unique proteins were found in syntenic groups, with two large regions corresponding to Prophage CP4-57 (region I) and proteins involved in lipid A biosynthesis (region II) (Fig. 4A; Additional file 1: Table S15). Lipid A modifications are important in modulating outer-membrane permeability, resistance to antimicrobial peptides, and host recognition [48], whereas prophage CP4-57 has been implicated in acid resistance [49], biofilm formation, and lactate utilization [50], suggesting that these elements may contribute to enhanced fitness of this isolate. An additional group of 5 syntenic proteins (01601-01605; Fig. 4B; Additional file 1: Table S15) was also unique to ESOS44-1, with domain analysis showing 01601 to contain the *N*-terminal part of LprI, a protein within *Mycobacterium tuberculosis* that detoxifies lysozyme [51].

Next, the presence of these 60 proteins unique to ESOS44-1 against the other *C. concisus* isolates was investigated in *C. rectus* ESOS44-4. A flavin reductase (00170), three proteins within region I, and notably, the putative lysozyme-detoxifying LprI homolog 01601 (00928 in *C. rectus*), were conserved in the proteome of ESOS44-4. Thus, the susceptibility of a subset of the isolates to lysozyme was then tested, and no differences were found between strains possessing LprI_01601 and those that do not (Fig. 4C). A modified lysozyme susceptibility protocol for Gram-negative bacteria including lactoferrin [52] was also applied, and again, no differences were observed (Fig. 4C), suggesting this protein may have other activities beyond lysozyme detoxification. Next, a mutant lacking LprI_01601 was generated using allelic exchange through natural transformation, and the ability of the mutant to survive intracellularly in primary macrophages was compared to the wild-type strain as well as representative GS2 strains. A significant decrease (~ 2.7-fold, *p* = 0.020; ANOVA-Dunnett’s test) in intracellular levels of the LprI_01601 mutant was found when compared to the wild-type ESOS44-1 (Fig. 4D). This decrease in intracellular levels was validated in the ESOS44-4 LprI_00928 mutant (Fig. 4D), showing a 1.9-fold decrease in levels. The addition of latrunculin A, an inhibitor of phagocytosis, abolished intracellular bacteria in both wild-type and mutant, indicating that internalization was completely reliant on phagocytosis and not transcellular invasion. Given this, lysosome acidification was tracked upon infection using LysoTracker. Acidification was more evident at 4 h in primary macrophages infected with ESOS44-1 wild-type and mutant strains, and this was not observed in the representative GS2 strain ESOS13-1 (Fig. 4E). This suggested that not only improved intracellular fitness but also increased phagocytosis contribute to the ESOS44-1 phenotype.

To provide insights into possible interacting partners of LprI_01601 within the host, recombinant LprI_01601 was used as a bait to pull down binding partners from primary human macrophage cell lysates (Fig. 4F). Ten human proteins that passed a strict filtration cutoff were identified, including the three lysosomal proteins cathepsin S, legumain, and galectin 9 (Fig. 4G), supporting a potential role for this bacterial protein within the host lysosome. Of note, the genes encoding cathepsin S and galectin 9, but not legumain were significantly upregulated in the transcriptome of patients with MET in our cohort (Additional file 1: Table S2).

The prevalence of LprI_01601 was then assessed in the shotgun metagenomics data set [10], similar to what was performed for TehA. A stepwise increase in prevalence was found (Fig. 4H), and the relationship with the disease cascade showed a borderline non-significant trend for this protein (*χ*^2^ = 3.27, *P* = 0.071; chi-square test for trend). Next, to conclusively detect the presence of *Campylobacter* with increased intracellular fitness, the prevalence of either “LprI_01601 or TehA” as well as either “LprI_01601 or the TehA operon” was also assessed in this data. A significant trend was identified for LprI_01601/TehA (*χ*^2^ = 4.52, *P* = 0.033; Additional file 2: Figure S8B) and a borderline non-significant trend identified for 01601/TehA operon (*χ*^2^ = 2.69, *P* = 0.10; Additional file 2: Figure S8C).

These findings show that certain *C. concisus* strains have increased intracellular fitness arising from specific genomic features, some of which are shared by *C. rectus.* These features increased in prevalence in shotgun sequencing data from diseased samples from an independent cohort.

### *Campylobacter* isolates with increased intracellular survival have differential effects on primary esophageal epithelial cells

**Fig. 5:**
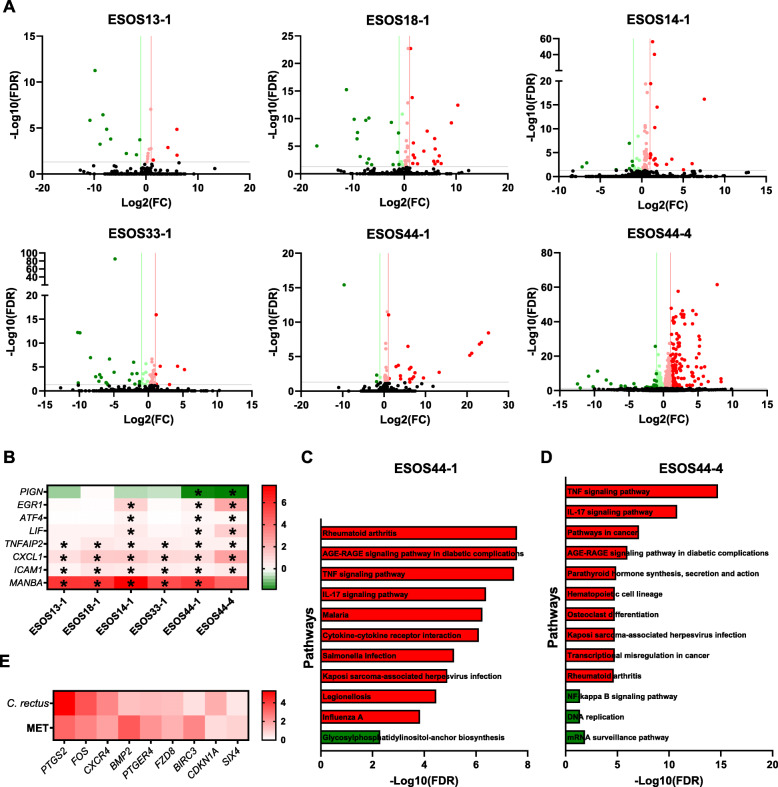
Host transcriptome changes of primary esophageal epithelial cells upon co-culture with *Campylobacter* isolates. Shotgun sequencing of mRNA was performed on a NovaSeq platform (*n* = 3 per condition except for ESOS44-1, *n* = 5). **A** Volcano plots of differentially expressed genes between negative controls and the different *Campylobacter* isolates tested. Differential expression was calculated using DESeq2. Red, upregulated upon infection, Green, downregulated upon infection. **B** Genes that showed distinct significant differential regulation across the different isolates tested. Scale bar is log_2_(fold-change). Patterns of particular interest included genes regulated only by ESOS44-1 and ESOS44-4 as well as those consistently regulated by ESOS44-4 and most *C. concisus* GS1. **C** Top ten pathways found to be significantly upregulated (red) and downregulated (green) following co-culture with ESOS44-1. **D** Top ten pathways found to be significantly upregulated (red) and downregulated (green) following co-culture with ESOS44-4. All analyses were conducted using Enrichr and *P* values were corrected for FDR. Only 1 and 3 pathways were identified to be significantly downregulated in ESOS44-1 and ESOS44-4 infection, respectively. **E** Genes found within KEGG “Pathways in cancer” and “Transcriptional misregulation in cancer” that were significantly upregulated by *C. rectus* ESOS44-4 and significantly upregulated in patients with metaplasia (MET). Only genes found to have > 0.5 log_2_(fold-change) in both conditions are presented

To investigate the effects of these different *Campylobacter* isolates on epithelial cells, primary esophageal epithelial cells were cultured and infected with representative isolates GS2 (ESOS13-1, ESOS18-1), GS1 (ESOS14-1, ESOS33-1, ESOS44-1), and *C. rectus* ESOS44-4. Bulk shotgun sequencing of RNA from the infected cells was performed and compared to that from non-infected cells. *C. concisus* isolates had a modest effect (*n* = 21 to 83 genes) when compared to *C. rectus* (*n* = 1028 genes) (Fig. 5A; Additional file 1: Tables S16-21). Several genes were found to be significantly regulated across all isolates tested including *TNFAIP2*, *CXCL1*, and *ICAM1*, while *MANBA* (Beta-mannosidase) was found to be significantly upregulated in all *C. concisus* isolates (Fig. 5B). The genes *EGR1*, *ATF4*, and *LIF* were upregulated by most GS1 isolates and *C. rectus* whereas *PIGN* was downregulated only in the isolates with especially high intracellular survival (ESOS44-1 and ESOS44-4) (Fig. 5B). Notably, *EGR1* was one of the 4 genes to be consistently, significantly upregulated in patients with GERD and MET (Fig. 1E).

Pathway analysis identified a substantial number of pathways commonly regulated by the different *C. concisus* isolates including several inflammatory pathways such as TNF and IL-17 signaling; however, “Glycosylphosphatidylinositol (GPI)-anchor biosynthesis” was uniquely downregulated by ESOS44-1 (Fig. 5C). Notably, while *C. rectus* ESOS44-4 also upregulated TNF and IL-17 signaling, it significantly upregulated “Pathways in cancer” as well as “Transcriptional misregulation in cancer” (Fig. 5D). Genes within these two pathways that were upregulated by *C. rectus* were then assessed against genes upregulated in patients with MET (Fig. 1D), and 9 genes were found to have a significant log_2_(fold-change) > 0.5 in both conditions when compared to their relevant controls (Fig. 5E).

These results demonstrated that *Campylobacter* isolates with high intracellular levels could regulate a range of inflammatory pathways in primary esophageal epithelial cells, as well as modulate genes identified to be differentially expressed in patients with GERD and MET. Further, *C. rectus* significantly upregulated pathways of high relevance to cancer development.

## Discussion

Little is known about the esophageal microenvironment when compared to the oral and lower gut. Here, we first assessed the host transcriptome and found that while some changes occur in GERD, most of the identified changes in expression, splicing, circular RNAs, and fusion events occur either in the transition from GERD to MET, or in MET. We then compared matched oral and esophageal microbiomes in the same cohort and confirmed that the esophageal microbiota is distinct, but most esophageal species can be source traced to the oral cavity. We identify changes in the esophageal microbiota with GERD and MET, with enrichment of *Campylobacter* found to be a consistent signature that starts in GERD. The microbiome and transcriptome profiles within the same samples were correlated, and the relative abundance of *Campylobacter* was associated with molecules associated with recognition of bacteria and the lysosome. In parallel, we isolated *Campylobacter* from the same patients and showed that isolates from patients with disease were more likely to have genomic features associated with intracellular fitness within primary immune cells. These genomic features were found to progressively increase in prevalence with disease progression in shotgun metagenomics data of esophageal brushings from an independent cohort. Co-culture of isolates with primary esophageal epithelial cells showed that most isolates that can survive intracellularly upregulated *EGR1*, which was found to be consistently upregulated in GERD and MET. *C. rectus* ESOS44-4 was also shown to upregulate pathways of high relevance to cancer development.

Bulk transcriptome profiling was suggestive of patients with metaplasia having higher levels of active mast cells, in contrast to higher levels of resting mast cells in patients with normal esophagi and GERD. This is concordant with immunohistochemical analysis of esophageal biopsies from patients with BAR which showed higher numbers of mast cells when compared to patients with GERD [53]. Interleukin-13, a cytokine commonly produced by mast cells, has been robustly linked to hyperplasia and mucus hypersecretion in airway epithelial cells [54, 55]. The transcriptome of patients with metaplasia was also found to resemble that of colonic cells, in line with the pathological transformation from a stratified epithelium to intestinal-like columnar cells. Robust markers of metaplasia in our study included cathepsin E (CTSE), which has been associated with gastric and colon cancers and previously shown to be upregulated in patients with BAR and EAC [56], as well as the anion/bicarbonate channel CFTR and PDZ-domain-containing mucins MUC17, MUC3A, and MUC12. The link between CFTR and MUC3 has been suggested to be involved in the mucus phenotype of certain diseases [57].

We also identified several markers that were consistent across patients with GERD and MET when compared to patients with normal biopsies. FREM2, a protein associated with alteration of the extracellular matrix to enable cell migration and rearrangements [58], showed a progressive increase from NORM to GERD to MET. This protein has also been suggested to facilitate IL-1β signal transduction through stabilizing IL-1R1 [59], an inflammatory pathway strongly linked to the EAC cascade [9]. Another marker of interest was the circular RNA transcript of CUL6, a component of the SCF ubiquitin ligase, which significantly decreased in prevalence in both GERD and MET and has been shown to promote defense against microbial species [60]. Of most relevance, however, was the progressive decrease in expression of *NAPSB*, a lysosomal aspartic protease related to napsin A (NAPSA) which has pseudogenized in most human populations (0–4.5% retain the active allele) [61].

We observed an increase in relative abundance of *Campylobacter* taxa at the stage of GERD that continued in metaplasia, regardless of the cohort studied or chemistry used. We also observed a significant increase in prevalence of a *Campylobacter* taxon of interest in disease. Several *Campylobacter* species such as *C. rectus* and *C. concisus* are members of the human oral microbiota but have been associated with various diseases. These bacteria are normally described as mucosa associated as they can traverse the mucus layer of the gastrointestinal tract efficiently [62]. They are also phylogenetically related to *Helicobacter pylori*, a causative agent of gastric cancer [63]. Their enrichment appeared to be at the expense of *Streptococcus* and *Herbaspirillium*. While the latter is considered a common extraction kit contaminant, viable *Herbaspirillium* have been previously isolated from the respiratory tract of humans [64]. Intriguingly, the relative abundance of *Campylobacter* was found to be in a co-exclusion relationship with *NAPSB*, one of the five genes consistently regulated in GERD and MET, which suggests *Campylobacter* may have some intracellular component to its lifestyle in vivo.

Isolation of *Campylobacter* species from this cohort confirmed intracellular survival within primary macrophages was a highly relevant characteristic across strains, with a genome-wide analysis identifying a genomic basis to this feature. Specifically, *C. concisus* isolates classified as GS1 had higher survival, and proteins unique to GS1 were enriched for oxidoreductases. A unique group of six syntenic proteins including tellurite resistance protein TehA was a key differentiating feature across these groups and was also found in *C. rectus* ESOS44-4. TehA has been found to contribute to the intracellular survival of *Corynebacterium diphtheriae* in epithelial cells [65] and resistance to antiseptics and disinfectants in *Escherichia coli* [66]. Of note, bacteria in the tumor microenvironment have been recently reported to consist mostly of species that have an intracellular niche in different tumor cell types including immune cells [67], with *Campylobacter* species being members of a polymicrobial signature associated with colorectal cancer [68].

A deeper analysis of one GS1 *C. concisus* isolate from a patient with MET that had higher relative survival than other GS1 isolates found one large unique genomic region linked to lipid A modifications as well as a prophage region previously linked to acid resistance [49]. A higher resistance to acid could contribute to this isolate having higher survival despite leading to higher lysosomal acidification upon infection. Further comparative analysis taking into consideration *C. rectus* isolated from the same patient pointed towards another group of syntenic proteins that included a protein containing a domain from LprI, a *Mycobacterium* protein that detoxifies lysozyme [51]. Knock-out of LprI_01601 did lead to decreased intracellular survival, and this finding was validated with the *C. rectus* LprI_00928 mutant, but the mechanism appeared to be independent of lysozyme. Indeed, on preliminary screen, recombinant LprI_01601 did not appear to interact with human lysozyme but was pulled down along with the lysosomal cysteine protease cathepsin S, galectin 9 [69], and legumain, of which the former is a substrate [70]. Notably, cathepsin S and galectin 9 were upregulated in patients with MET in our cohort. Lower cathepsin S has been previously linked to higher intracellular survival of *Mycobacterium tuberculosis* in macrophages [71], and this autophagy-associated protein has been linked to inflammatory and infectious periodontal disease [72]. However, the influence of knock-out of LprI_01601 on the remainder of the syntenic proteins cannot be discounted. Two of these proteins are acetyltransferases, a function that influences bacterial virulence [73, 74], most notably, the capacity of *C. jejuni* for lysozyme resistance and intracellular survival in macrophages through acetylation of its peptidoglycan [57].

Of significance, the identified signatures delineating *Campylobacter* with increased intracellular fitness (TehA operon and LprI_01601) increased in prevalence with disease progression (normal on PPIs, GERD, then MET) in shotgun metagenomics data from esophageal brushings of patients with GERD and MET, supporting a possible role for *Campylobacter* isolates possessing these proteins in esophageal disease. These results are of biological relevance given the unsupervised and unbiased nature of this analysis.

Co-culture of representative GS2 and GS1 isolates as well as *C. rectus* ESOS44-4 with primary esophageal epithelial cells showed that all isolates could induce TNF and IL-17 signaling but only the GS1 isolates (ESOS14-1 and ESOS44-1) and *C. rectus* led to an upregulation of *EGR1*, a gene that was also upregulated in patients with GERD and MET. This gene has been reported to be potential biomarker of BAR with low-grade dysplasia and EAC [75], and to have high expression in precancerous lesions of the stomach and esophagus [76, 77]. These isolates also upregulated *LIF*, an IL-6 family cytokine associated with the lack of efficacy of neoadjuvant therapy in EAC [78]. Another gene of interest is *PIGN*, found to be downregulated by the two isolates ESOS44-1 and ESOS44-4 with increased intracellular levels. *PIGN* has been reported to be a chromosomal instability suppressor in cancer whose silencing can lead to DNA replication stress and associated damage [79]. Importantly, *C. rectus* infection significantly upregulated > 50 genes associated with cancer development, and a range of these were commonly upregulated in patients with MET as well. Given that *C. rectus* is substantially lower in prevalence in the human oral cavity than the commonly detected *C. concisus*, the specific consequences of its presence within the microbiota on the host need to be investigated further.

Our study had some limitations. This includes the size of the prospective cohort which we attempted to overcome by screening for signatures in a previously published cohort. Future studies should validate the presence of these signatures in larger cohorts and take into account additional variables such as smoking and diet. Further, we did not include patients with EAC as our design focused on etiological factors of metaplasia. Additional studies should investigate if these signatures are present in EAC, or if similar to gastric cancer, microbial etiological factors disappear.

## Conclusions

Taken together, our work utilizes multi-omics strategies to identify strain-level signatures of relevance to esophageal disease, pointing towards a role for *C. rectus* and some *C. concisus* GS1 with increased intracellular fitness in primary macrophages. Our work highlights the utility of an unbiased systems approach in transitioning complex microbiome signatures from correlation to causation in situations where animal models are not readily available.

## Supplementary Information

**Additional file 1: Table S1.** Transcripts differentially expressed between normal and GERD samples. **Table S2.** Transcripts differentially expressed between normal and MET samples. **Table S3.** Pathway analysis of differentially expressed transcripts. **Table S4.** Genes identified with possible differences in splicing events. **Table S5.** Intron retention and exon skipping events found between disease groups. **Table S6.** Circular RNAs tested for differential prevalence within disease groups. **Table S7.** Gene fusion events identified within the esophageal samples. **Table S8.** Per taxa differences between saliva and esophageal samples. **Table S9.** Per taxa differences between normal and GERD esophageal samples. **Table S10.** Per taxa differences between normal and MET esophageal samples. **Table S11.** Per taxa differences between normal and disease esophageal samples. **Table S12.** MINE analysis between *Campylobacter* and transcripts. **Table S13.** MINE analysis between *Campylobacter* iOTU36 and transcripts. **Table S14.** Conservation of proteins unique to GS1 *C. concisus* isolates. **Table S15.** Proteins unique to *C. concisus* ESOS44-1. **Table S16.** Differential expression between control and *Campylobacter* ESOS13-1. **Table S17.** Differential expression between control and *Campylobacter* ESOS14-1. **Table S18.** Differential expression between control and *Campylobacter* ESOS18-1. **Table S19.** Differential expression between control and *Campylobacter* ESOS33-1. **Table S20.** Differential expression between control and *Campylobacter* ESOS44-1. **Table S21.** Differential expression between control and *Campylobacter* ESOS44-4.

**Additional file 2: Figure S1.** Splicing events within the esophageal transcriptome. **Figure S2.** Exon skipping events within the esophageal transcriptome. **Figure S3.** Circular RNAs and gene fusion within the esophageal transcriptome. **Figure S4.** Saliva and esophageal microbiota in the early stages of the EAC cascade. **Figure S5.** Esophageal microbiota in the early stages of the EAC cascade. **Figure S6.** Procrustes analysis between transcriptome and microbiome. **Figure S7.** Correlations between *Campylobacter* iOTU36 and transcript counts. **Figure S8.** Prevalence of regions of interest in shotgun metagenomics data.

## Data Availability

The PacBio 16S rRNA amplicon sequencing data (https://www.ebi.ac.uk/ena/browser/view/PRJEB46879) [80], the Illumina 16S rRNA amplicon sequencing data (https://www.ebi.ac.uk/ena/browser/view/PRJEB46880) [81] and bacterial genome assemblies (https://www.ebi.ac.uk/ena/browser/view/PRJEB46877) [82] were submitted to the European Nucleotide Archive (ENA). The RNA sequencing data from esophageal epithelial cells was also submitted to ENA (https://www.ebi.ac.uk/ena/browser/view/PRJEB46795) [83]. The RNA sequencing data from esophageal biopsies are not publicly available to maintain patient confidentiality but are available from the corresponding author on reasonable request. In-house scripts for the identification of possible functional consequences of alternative splicing events were submitted to zenodo (10.5281/zenodo.5179440) [21]. The data from the previous cohort profiling esophageal brushings is available from ENA (16S rRNA amplicon: https://www.ebi.ac.uk/ena/browser/view/PRJEB25236; shotgun metagenomics: https://www.ebi.ac.uk/ena/browser/view/PRJEB25422) [10].

## Abbreviations

ESCC: Esophageal squamous cell carcinoma
EAC: Esophageal adenocarcinoma
GERD: Gastro-esophageal reflux disease
BAR: Barrett’s esophagus
MET: Metaplasia
NORM: Normal
BMI: Body mass index
OTU: Operational taxonomic unit
ANI: Average nucleotide identity
