# Supplementary material for: Multi-omics of the esophageal microenvironment identifies signatures associated with progression of Barrett’s esophagus
Source: Genome Med. 2021 Aug 19;13:133. doi: 10.1186/s13073-021-00951-6 (PMC8375061; doi:10.1186/s13073-021-00951-6)
Supplement: Supplementary file 2 — Additional file 2: Figure S1. Splicing events within the esophageal transcriptome. Figure S2. Exon skipping events within the esophageal transcriptome. Figure S3. Circular RNAs and gene fusion within the esophageal transcriptome. Figure S4. Saliva and esophageal microbiota in the early stages of the EAC cascade. Figure S5. Esophageal microbiota in the early stages of the EAC cascade. Figure S6. Procrustes analysis between transcriptome and microbiome. Figure S7. Correlations between Campylobacter iOTU36 and transcript counts. Figure S8. Prevalence of regions of interest in shotgun metagenomics data. [file 13073_2021_951_MOESM2_ESM.docx]

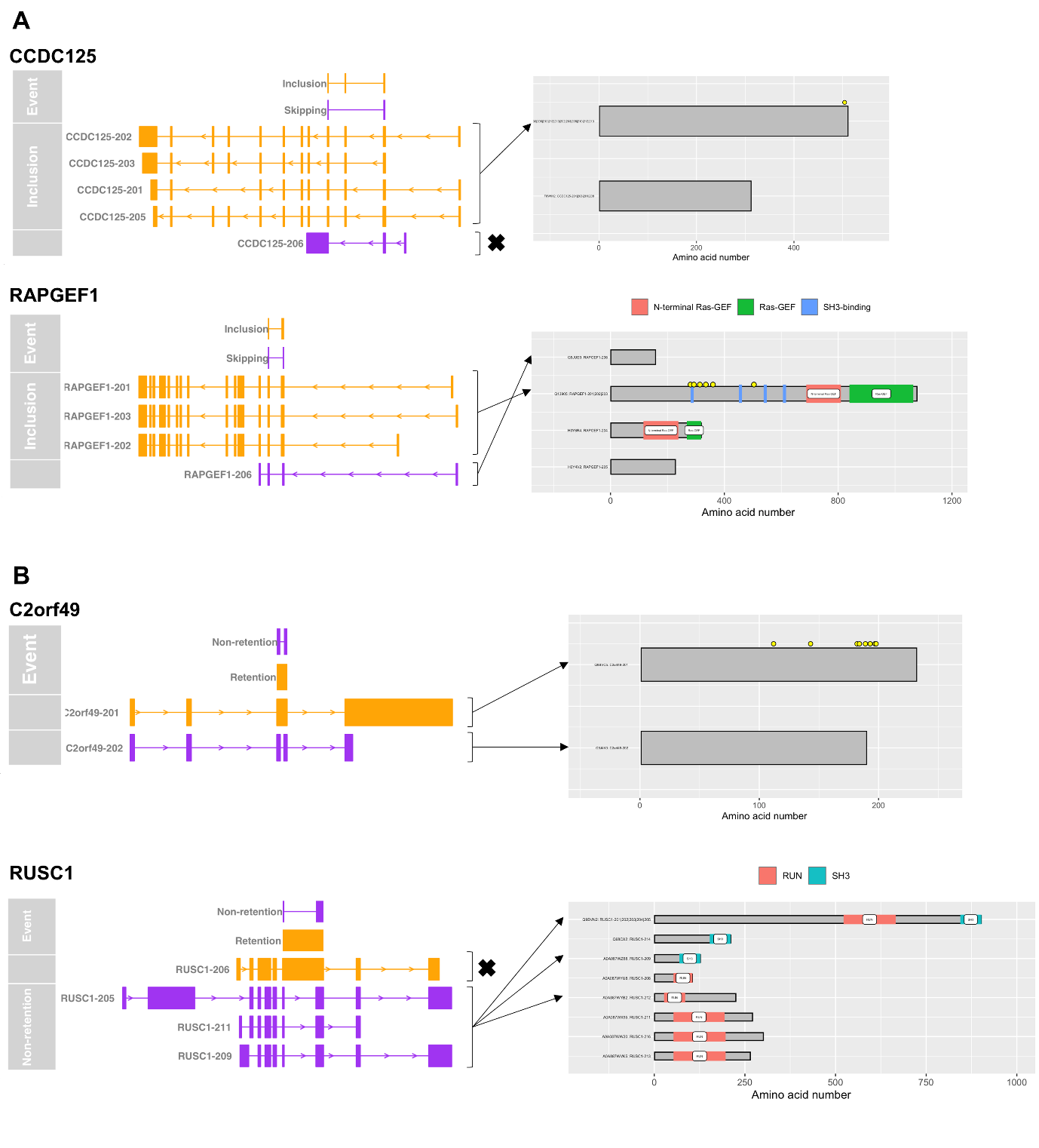


**Figure S1**

**Splicing events within the host esophageal transcriptome that lead to predicted protein-level changes between groups.** Splicing events were identified using rMATS. **A:** Significant splicing events identified when comparing NORM and GERD samples. Both events were exon skipping events. **B:** Significant intron retention events identified when comparing NORM and MET samples.


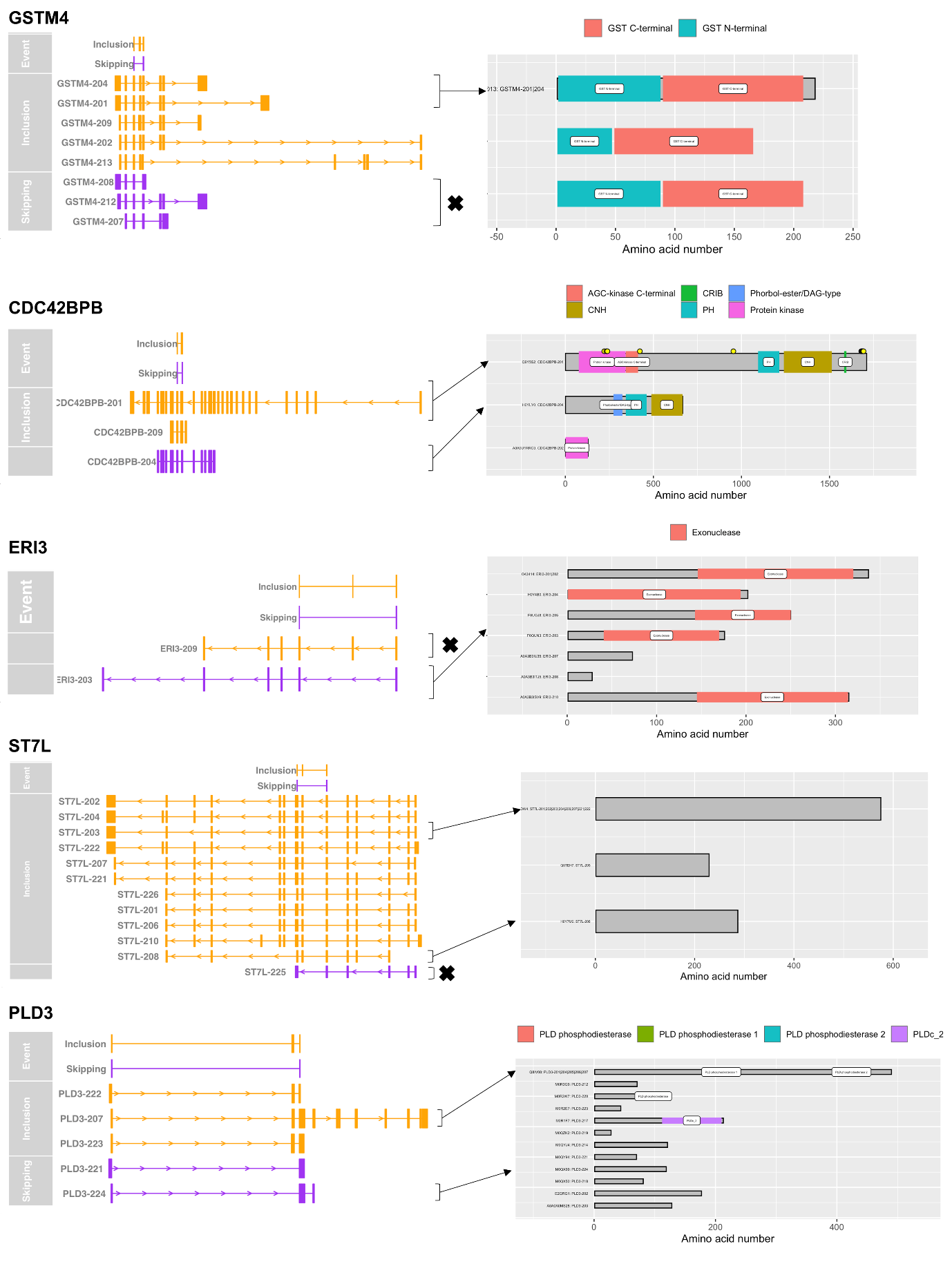


**Figure S2**

**Exon skipping events within the host esophageal transcriptome that lead to predicted protein-level changes between NORM and MET samples.** Splicing events were identified using rMATS.


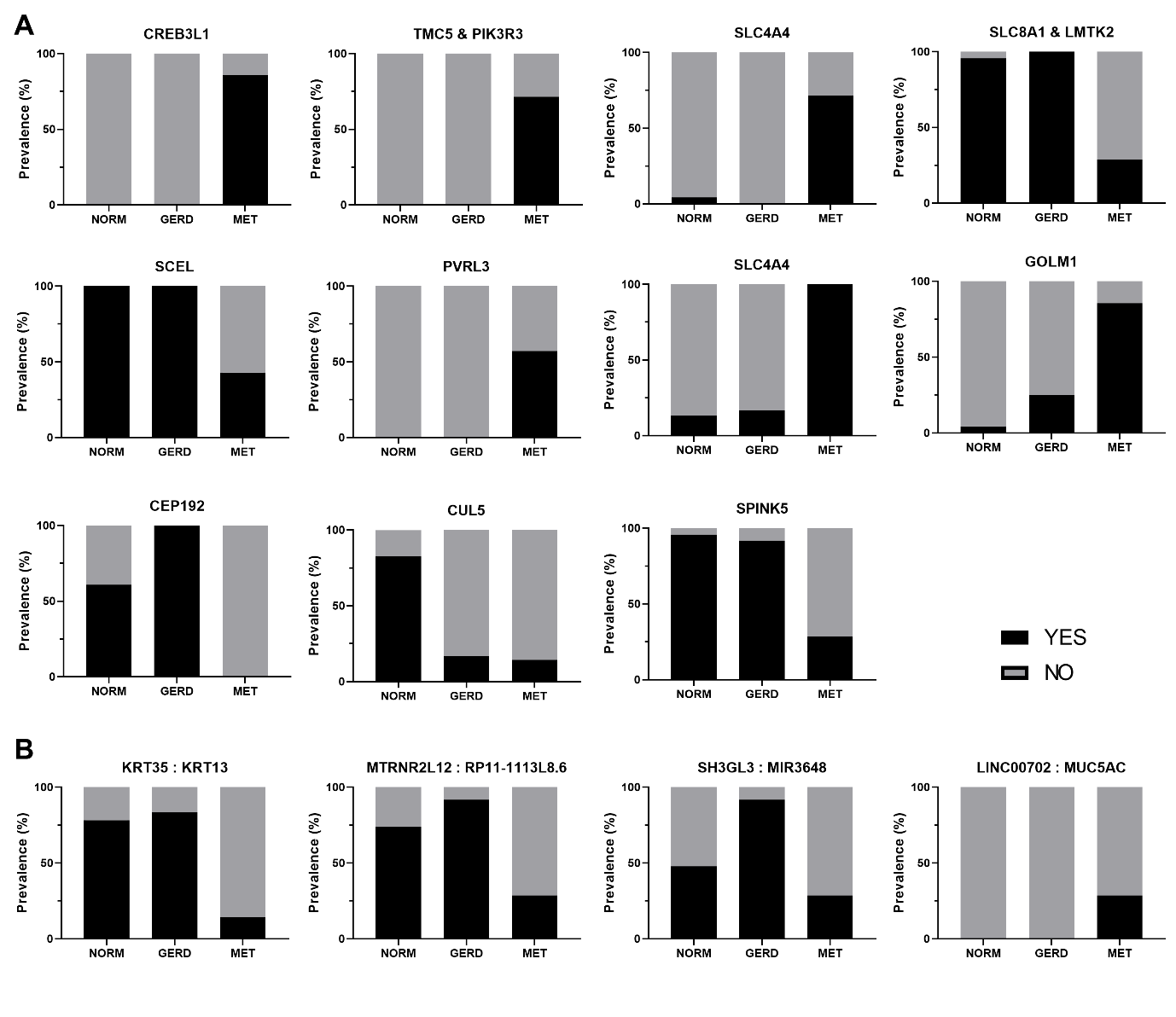


**Figure S3**

**Circular RNAs and gene fusion events within the host esophageal transcriptome that are differentially prevalent across the groups. A:** Circular RNAs found to be significantly different in prevalence across groups after multiple comparison correction. **B:** Gene fusion events found to be significantly different in prevalence across groups after multiple comparison correction.


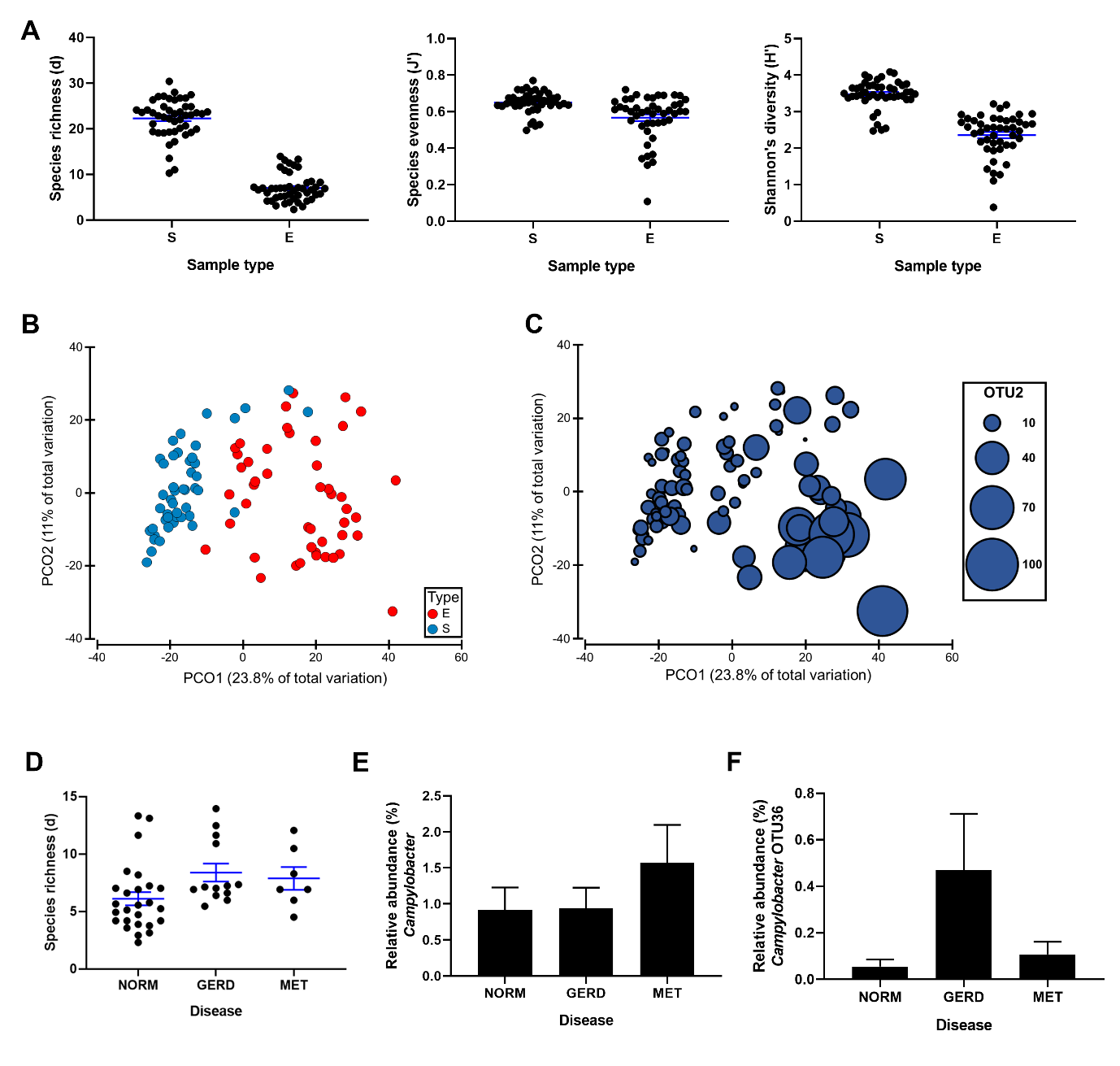


**Figure S4**

**The saliva and esophageal microbiota in subjects in the early stages of the EAC cascade.** The V4 region of 16S rRNA gene was sequenced on an Illumina platform. Sample E24 did not sequence to saturation, and thus, was excluded from further analysis along with its matching saliva sample (S24). **A:** Comparison of alpha diversity measures between saliva (S) and esophageal (E) samples. Significant differences between sample types (Richness: Pseudo-F: 404.4, P=0.001, df=90; Evenness: Pseudo-F: 16.4, P=0.001, df=90; Shannon’s diversity: Pseudo-F: 116.9, P=0.001, df=90) were observed across all measures using linear models that corrected for age, sex, PPI, BMI, reflux symptoms and disease. **B:** Principal coordinate analysis of Bray-Curtis resemblance matrix generated from square-root transformed iOTU relative abundances. Distance-based linear models corrected for all variables identified significant differences in composition between sample types (Pseudo-F: 18.8, P=0.001, df=90) and with age (Pseudo-F: 2.0, P=0.026, df=90). **C:** The same principal coordinate analysis as above incorporating the relative abundance of *Streptococcus* iOTU2. **D:** Esophageal species richness stratified according to disease. **E:** Mean relative abundance of *Campylobacter* stratified according to disease. **F:** Mean relative abundance of *Campylobacter* iOTU36 stratified according to disease. Errors are SEM.


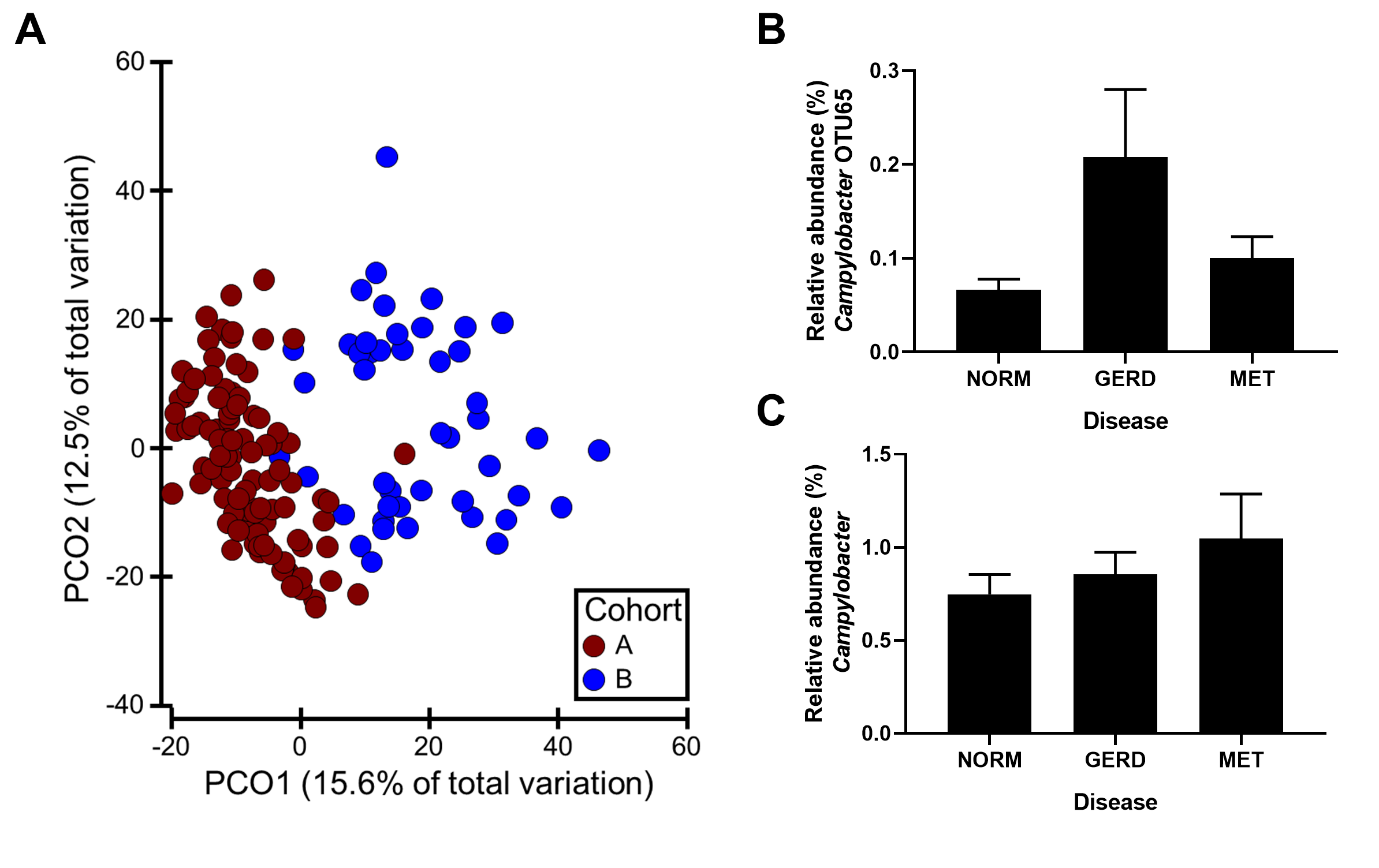


**Figure S5**

**The esophageal microbiota in subjects in the early stages of the EAC cascade.** The V4 region of 16S rRNA gene was sequenced on an Illumina platform. Data from the current prospective cohort and a previously published cohort (Table 1) were combined. **A:** Principal coordinate analysis of Bray-Curtis resemblance matrix generated from square-root transformed ciOTU relative abundances. A strong batch effect can be observed. **B:** Mean relative abundance of *Campylobacter* ciOTU65 stratified according to disease. ciOTU65 and iOTU36 correspond to the same consensus sequence. **C:** Mean relative abundance of *Campylobacter* stratified according to disease. Errors are SEM.


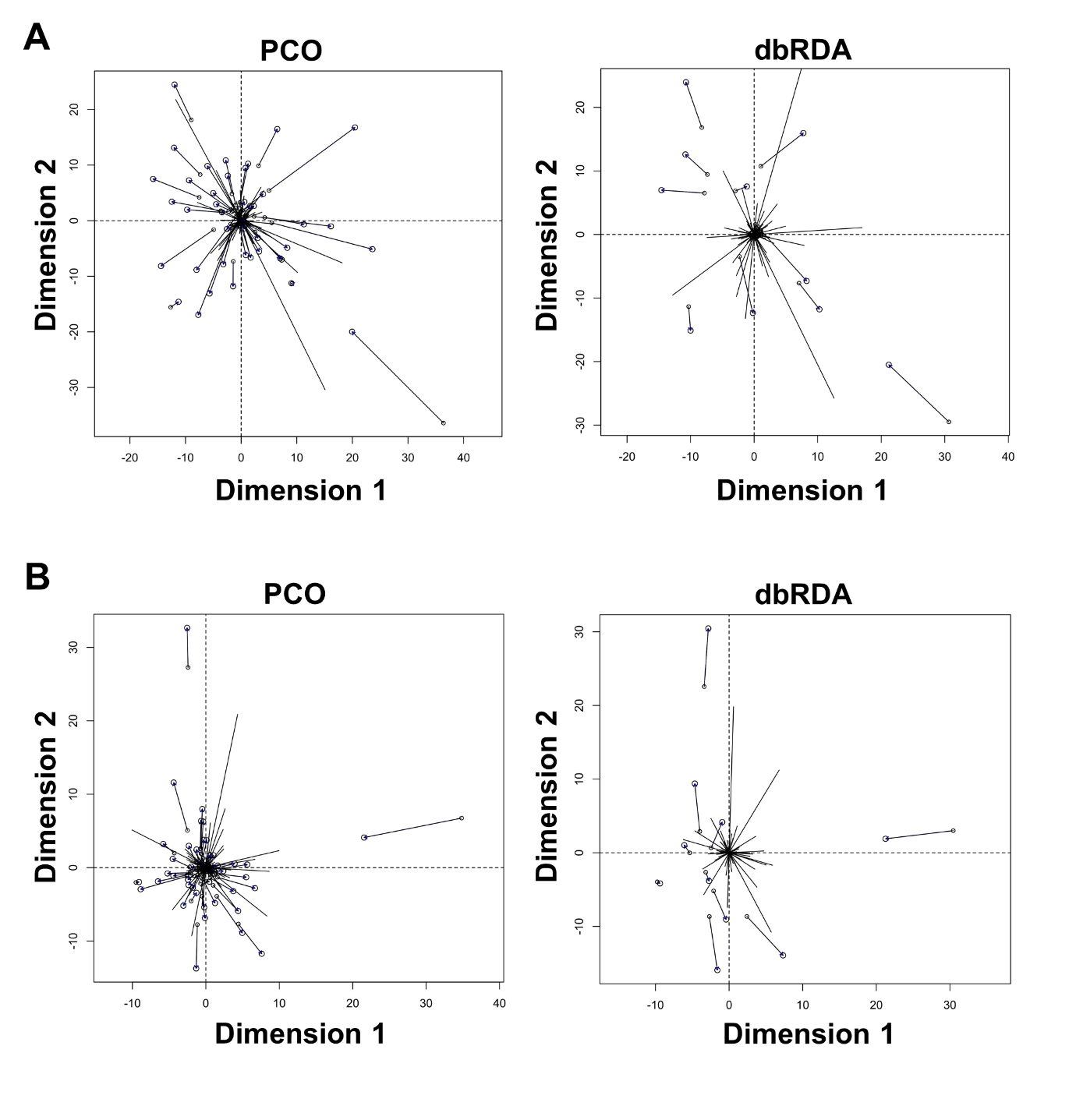


**Figure S6**

**Procrustes analysis between transcriptome and microbiome profiles.** Both principal coordinate axes (PCO) and distance-based redundancy axes (dbRDA) were tested. **A:** Ordinations from 16S rRNA amplicon sequencing data using PacBio. **B:** Ordinations from 16S rRNA amplicon sequencing using Illumina.

**
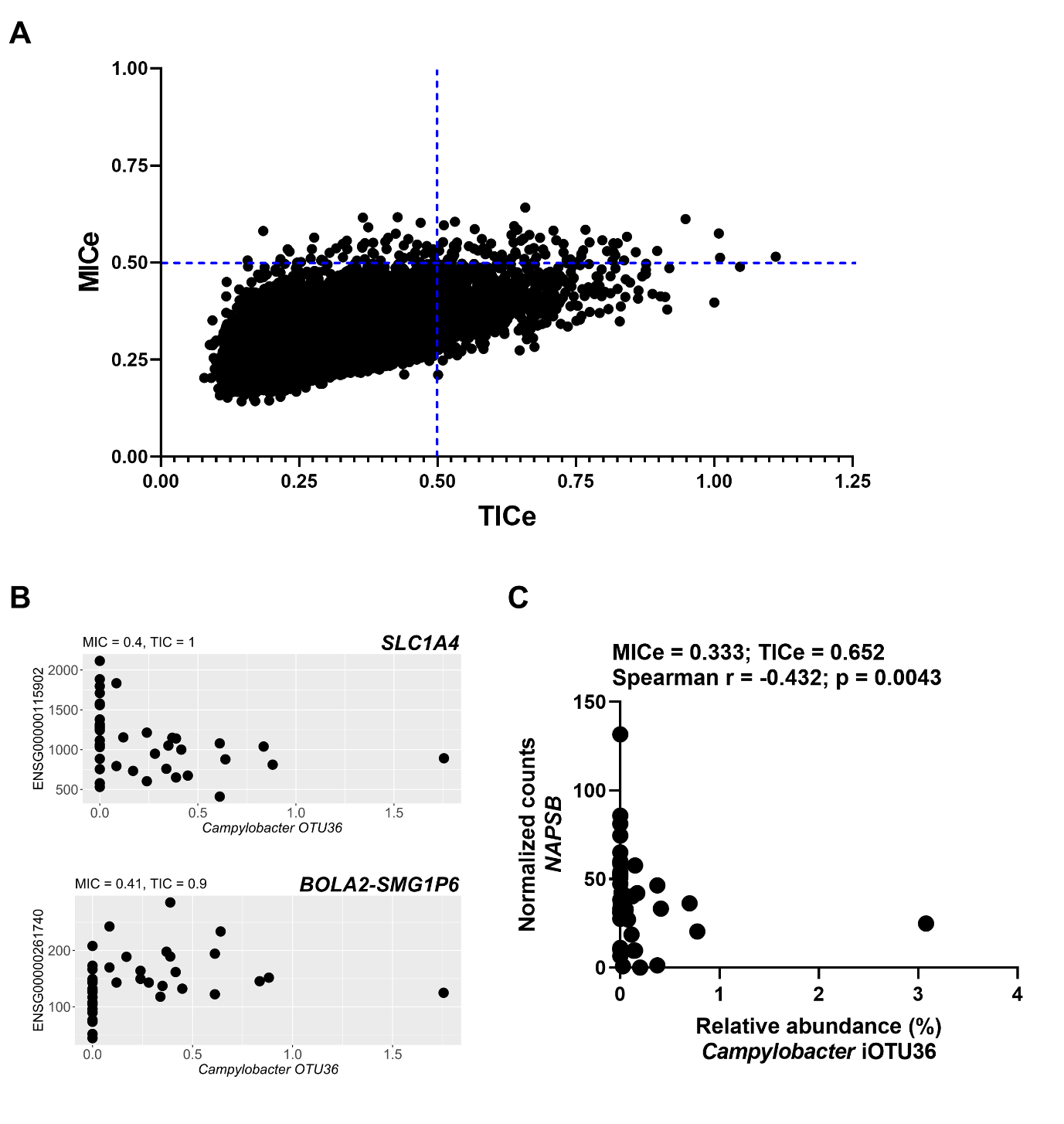
**

**Figure S7**

**Non-parametric correlations between *Campylobacter* iOTU36 and host transcript counts. A:** Scatter plot of MICe and TICe values from all correlations. **B:** Transcripts with high TICe values when correlated with *Campylobacter* iOTU36. **C:** Co-exclusion relationship between *NAPSB* transcripts and *Campylobacter* iOTU36 (illumina). Non-parametric correlations were identified through MINe and confirmed with Spearman’s correlation.


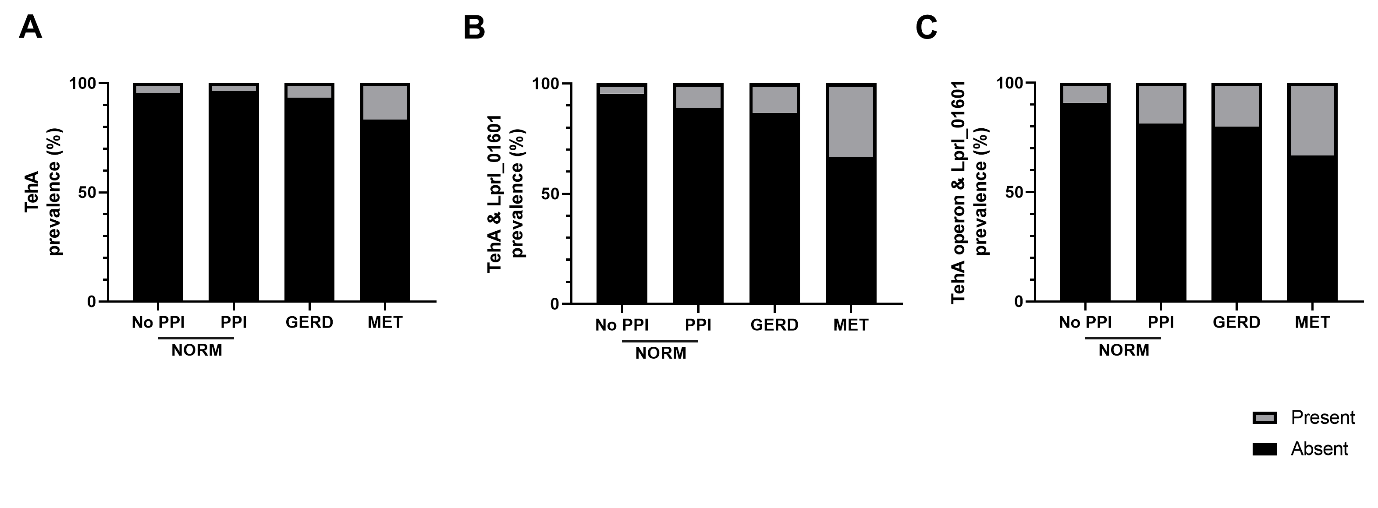


**Figure S8**

**Prevalence of regions of interest in shotgun metagenomics data of esophageal mucosal brushings from patients in the early stages of the EAC cascade. A:** Prevalence of the TehA gene. **B:** Prevalence of either TehA gene or LprI_01601. **C:** Prevalence of either TehA operon or LprI_01601. Reads aligning to the regions were identified using BWA-MEM.
